# Inflammatory and tolerogenic myeloid cells determine outcome following human allergen challenge

**DOI:** 10.1084/jem.20221111

**Published:** 2023-07-10

**Authors:** Astrid L. Voskamp, Tamar Tak, Maarten L. Gerdes, Roberta Menafra, Ellen Duijster, Simon P. Jochems, Szymon M. Kielbasa, Tom Groot Kormelink, Koen A. Stam, Oscar R.J. van Hengel, Nicolette W. de Jong, Rudi W. Hendriks, Susan L. Kloet, Maria Yazdanbakhsh, Esther C. de Jong, Roy Gerth van Wijk, Hermelijn H. Smits

**Affiliations:** 1 Department of Parasitology, Leiden University Medical Center, Leiden, Netherlands; 2 Department of Ear, Nose and Throat, Erasmus University Medical Center, Rotterdam, Netherlands; 3 Leiden Genome Technology Center, Leiden University Medical Center, Leiden, Netherlands; 4 Department of Internal Medicine, Section Allergology and Clinical Immunology, Erasmus University Medical Center, Rotterdam, Netherlands; 5 Department of Biomedical Data Sciences, Leiden University Medical Center, Leiden, Netherlands; 6 Department of Exp Immunology, Amsterdam University Medical Centers, Amsterdam, Netherlands; 7 Department of Pulmonary Medicine, Erasmus University Medical Center, Rotterdam, Netherlands

## Abstract

Innate mononuclear phagocytic system (MPS) cells preserve mucosal immune homeostasis. We investigated their role at nasal mucosa following allergen challenge with house dust mite. We combined single-cell proteome and transcriptome profiling on nasal immune cells from nasal biopsies cells from 30 allergic rhinitis and 27 non-allergic subjects before and after repeated nasal allergen challenge. Biopsies of patients showed infiltrating inflammatory HLA-DR^hi^/CD14^+^ and CD16^+^ monocytes and proallergic transcriptional changes in resident CD1C^+^/CD1A^+^ conventional dendritic cells (cDC)2 following challenge. In contrast, non-allergic individuals displayed distinct innate MPS responses to allergen challenge: predominant infiltration of myeloid-derived suppressor cells (MDSC: HLA-DR^low^/CD14^+^ monocytes) and cDC2 expressing inhibitory/tolerogenic transcripts. These divergent patterns were confirmed in ex vivo stimulated MPS nasal biopsy cells. Thus, we identified not only MPS cell clusters involved in airway allergic inflammation but also highlight novel roles for non-inflammatory innate MPS responses by MDSC to allergens in non-allergic individuals. Future therapies should address MDSC activity as treatment for inflammatory airway diseases.

## Teaser

Innate mononuclear phagocytic cells (MPS) in the nasal mucosa are the first to encounter inhaled allergens. MPS from allergic rhinitis patients showed a pro-inflammatory response during experimental allergen challenge, while clinically silent non-allergic controls displayed a tolerizing/anti-inflammatory response.

## Introduction

Innate cells comprosing the mononuclear phagocytic system (MPS) contribute to tissue homeostasis by phagocytosing cellular debris and pathogens. After phagocytosis, they can present antigens to cells of the adaptive immune system and through specific co-stimulatory signals determine whether a tolerogenic or inflammatory response is initiated (Bassler et al., 2019; Murray et al., 2014). Furthermore, MPS cells can determine whether an antiviral/bacterial T helper (Th)1/Th17 response or an anti-parasitic/allergic Th2 response is initiated.

Allergic airway diseases, such as allergic rhinitis (AR) or allergic asthma, are characterized by sensitization to harmless allergens upon inhalation, disturbance of local immune homeostasis, and a Th2 immune response (Boonpiyathad et al., 2019). Studies with repeated grass and tree pollen challenge showed a rapid influx of monocytes into nasal tissue of AR patients, preceding Th2 cells and eosinophils, suggesting an early inflammatory role for MPS cells (Eguíluz-Gracia et al., 2016). On the other hand, MPS can induce tolerance by production of anti-inflammatory molecules such as retinoid acid, TGFβ, and IL-10 (de Waal Malefyt et al., 1991; Domogalla et al., 2017; Said et al., 2010; Xu et al., 2006).

Much of our knowledge on human MPS cells has been derived from peripheral blood. However, conventional dendritic cell (cDC)2, monocytes, and macrophages (MF) are all known to receive tissue-specific priming and inflammatory signals, leading to differences in appearance and functionality (Balan et al., 2019; Gordon and Pluddemann, 2017). Furthermore, many MF populations are seeded prior to birth and are independent of replenishment from the blood. Therefore, to gain understanding of airway epithelial and immune cells, single-cell analyses such as mass cytometry (CyTOF) and single-cell RNA sequencing (scRNA-Seq) have been employed on upper airway biopsies from healthy individuals as well as from patients suffering from, e.g., asthma and SARS-CoV2 infection (Ordovas-Montanes et al., 2018; Roukens et al., 2022; Schiller et al., 2019; Sungnak et al., 2020; Vieira Braga et al., 2019).

To understand the local nasal immune response to allergens, we performed extensive, single-cell proteome and transcriptome profiling on immune cells in nasal tissue of both AR and non-allergic subjects before and after controlled house dust mite (HDM) challenge. Our findings showed emerging inflammatory MPS cells at nasal mucosa imposing allergic symptoms in allergic individuals, while tolerogenic MPS cells are active in healthy individuals with clinically silent responses.

## Results

### AR subjects display a type 2 phenotype

**Figure 1.**
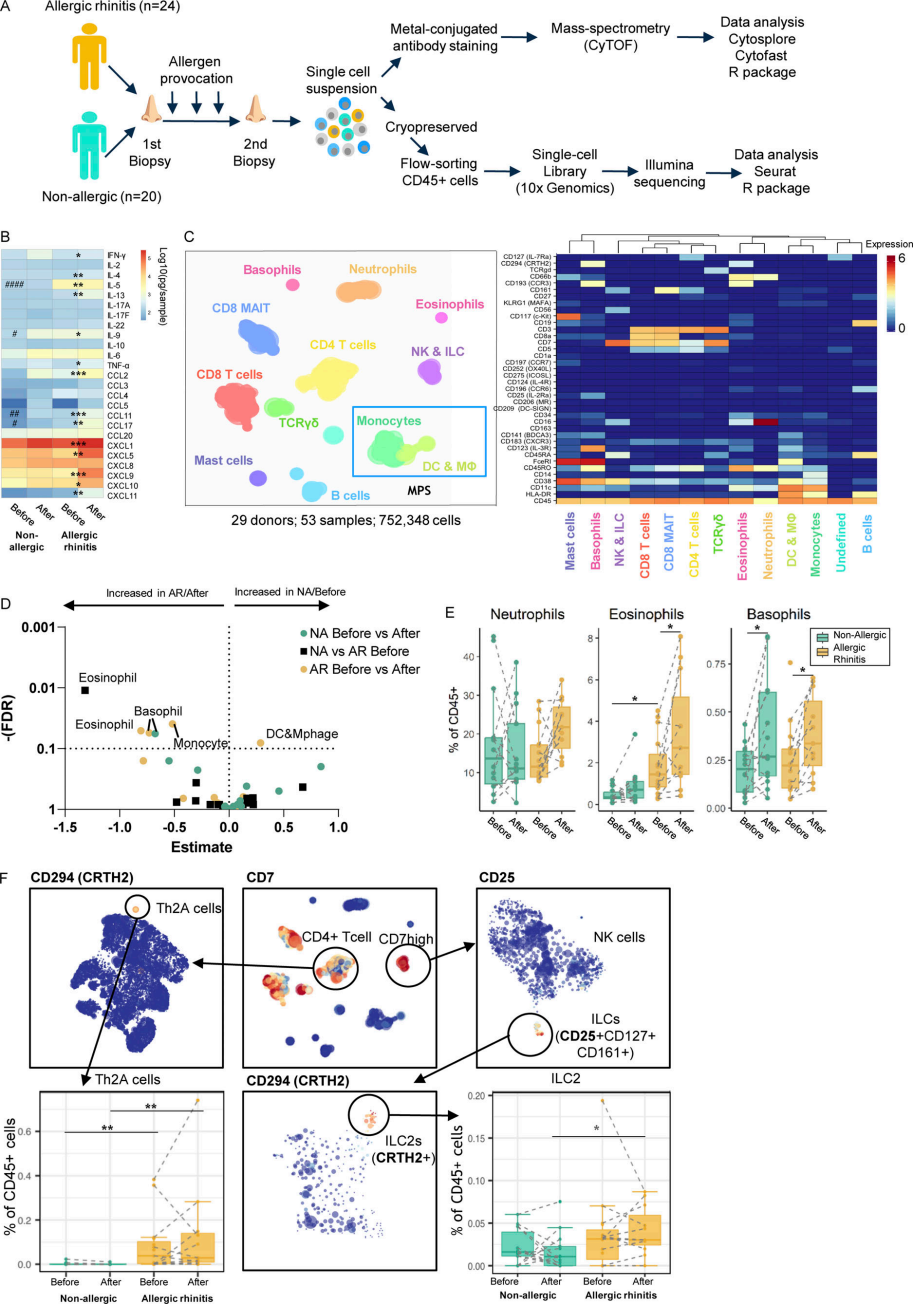
**Study setup, proteomic data clustering, and lineage identification. (A)** Schematic of study design and protocol for single-cell transcriptomic and proteomic data collection and analysis of nasal biopsy cells from allergic and non-allergic individuals. **(B)** Heatmap of nasal fluid cytokine contents. **(C)** Hierarchical stochastic neighbor embedding plot of cell lineages identified in proteomic data and heatmap of markers used. MPS cells, based on HLA-DR and CD11c or CD123, are indicated in the blue box within the Hierarchical Stochastic Neighbor Embedding plot. **(D)** Volcano plot of statistical test results on the frequency of clusters identified in C. **(E)** Boxplots of cluster frequencies for clusters showing significant differences. **(F)** Subclustering data from C to identification and quantification of allergy-associated Th2A (CD3^+^ CD8^−^ CD45RO^−^ CD27^−^ CD161^+^ CRTH2^+^) and ILC2 (CD7^+^(CD3^−^) CD25^+^ CD127^+^ CD161^+^ CRTH2^+^). */#P < 0.05, **/##P < 0.01, ***P < 0.001, and ####P < 0.0001 between before/after (*) and between non-allergic vs. AR patients (#), as determined by Wilcoxon matched-pairs signed rank tests, or Mann-Whitney test where applicable (B and F) or by generalized linear mixed model (GLMM) with FDR correction (D and E). **(B)** 15 samples obtained from 12 non-allergic controls and 36 samples from 21 AR patients. **(D–F)** Data obtained from 14 non-allergic controls and 14 AR patients analyzed freshly (also see Table S10).

Biopsies and nasal secretions were obtained from non-allergic and AR subjects (Table 1) before and after 3 d of repeated HDM challenge (Fig. 1 A). Following the first nasal allergen challenge, symptom scores, including sneezing, itchy eyes, nasal congestion, and rhinorrhea, were recorded to monitor allergic responses. AR subjects responded to HDM challenge, with a group average summed symptom score of 18 (range 9–28; Lebel et al., 1988). Non-allergic subjects had symptom scores below 5 (the threshold for a positive response; Table 1). The clinical symptoms corroborated with changes in cytokine and chemokine levels measured in nasal secretion before and after allergen challenge: at baseline increased levels of IL-5, IL-9, CCL11 (eotaxin), and CCL17 (TARC) were found in allergic patients. Following allergen challenge, more IL-4, IL-5, IL-9, IL-13, TNFa, INF-γ, CCL11, CCL17, CXCL1, CXCL5, CXCL9, CXCL10, and CXCL11 were detected in AR subjects, while no significant differences were found in non-allergic individuals (Fig. 1 B).

**Table 1.** Cohort characteristics

		
CyTOF cohort	Non-allergic	AR
Number of subjects	14 (1 WD)	14 (4 WD)
Female (%)	64.3	46.7
Median age (min-max)	23 (18–49)	26 (18–45)
Median SPT	Negative (<0.4)	1.01 (0.47–3.47)
Median HDM-specific serum IgE (CAPFEIA)	Negative (<0.35 KU/I)	12.7 (0.39- >100)
Median symptom score	Negative (<5)	18 (11–28)
Co-sensitizations (median)	0	2.5
Birch pollen (%)	0	68.75
Grass pollen (%)	0	68.75
Cat (%)	0	56.25
Dog (%)	0	50
		
scRNA-Seq cohort	Non-allergic	AR
Number of subjects	18	21
Female (%)	61.1	52.4
Median age (min-max)	25 (19–49)	24 (18–45)
Median SPT	Negative (<0.4)	1.01 (0.46–2.48
Median HDM-specific serum IgE (CAPFEIA)	Negative (<0.35 KU/I)	8.9 (0.39–>100)
Median symptom score	Negative (<5)	18 (9–28)
Co-sensitizations (median)	0	3
Birch pollen (%)	0	75
Grass pollen (%)	0	81.25
Cat (%)	0	43.75
Dog (%)	0	56.25
		
Cohort overlap	12	12

**Figure S1.**
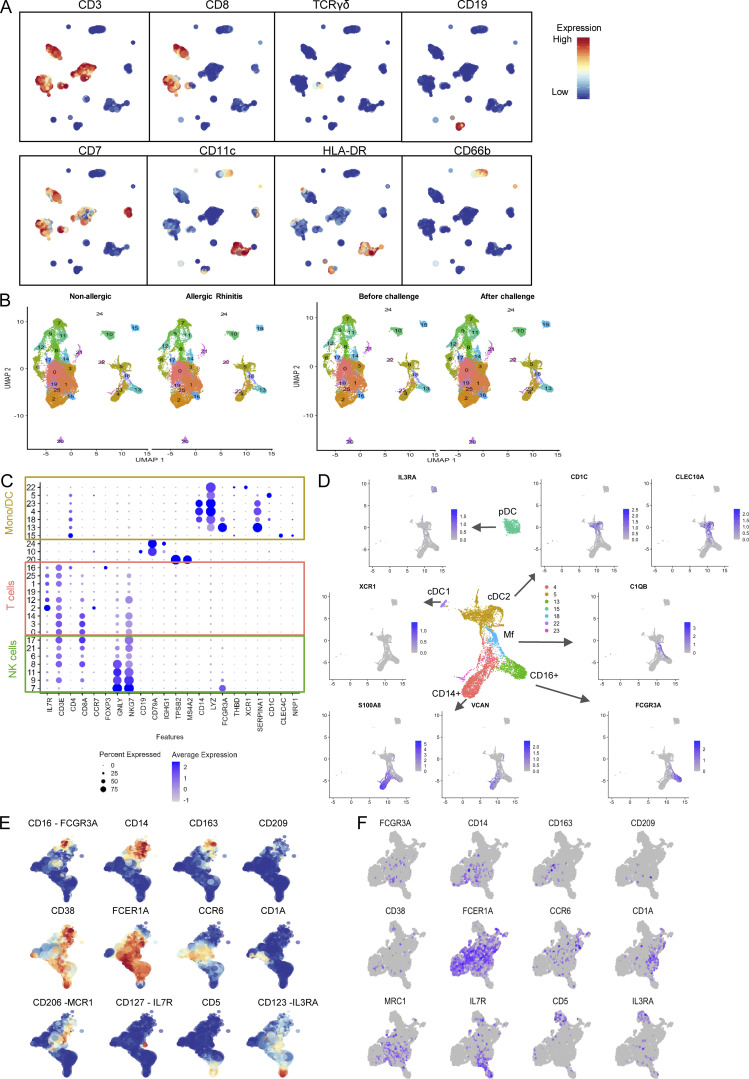
**Cytosplore clustering of proteomic data cells. (A)** Automated clustering was performed by hierarchical stochastic neighbor embedding using Cytosplore software. Cell clusters were identified based on known markers of major immune cell lineages. **(B)** Clustering by study group/timepoint. **(C)** Dotplot indicating expression of signature genes for different lineages per cluster. **(D)** Featureplots indicating expression of signature genes on UMAP. **(E and F)** Expression of signature genes in subclustered monocyte populations in (E) proteomic and (F) transcriptomic data.

**Figure 2.**
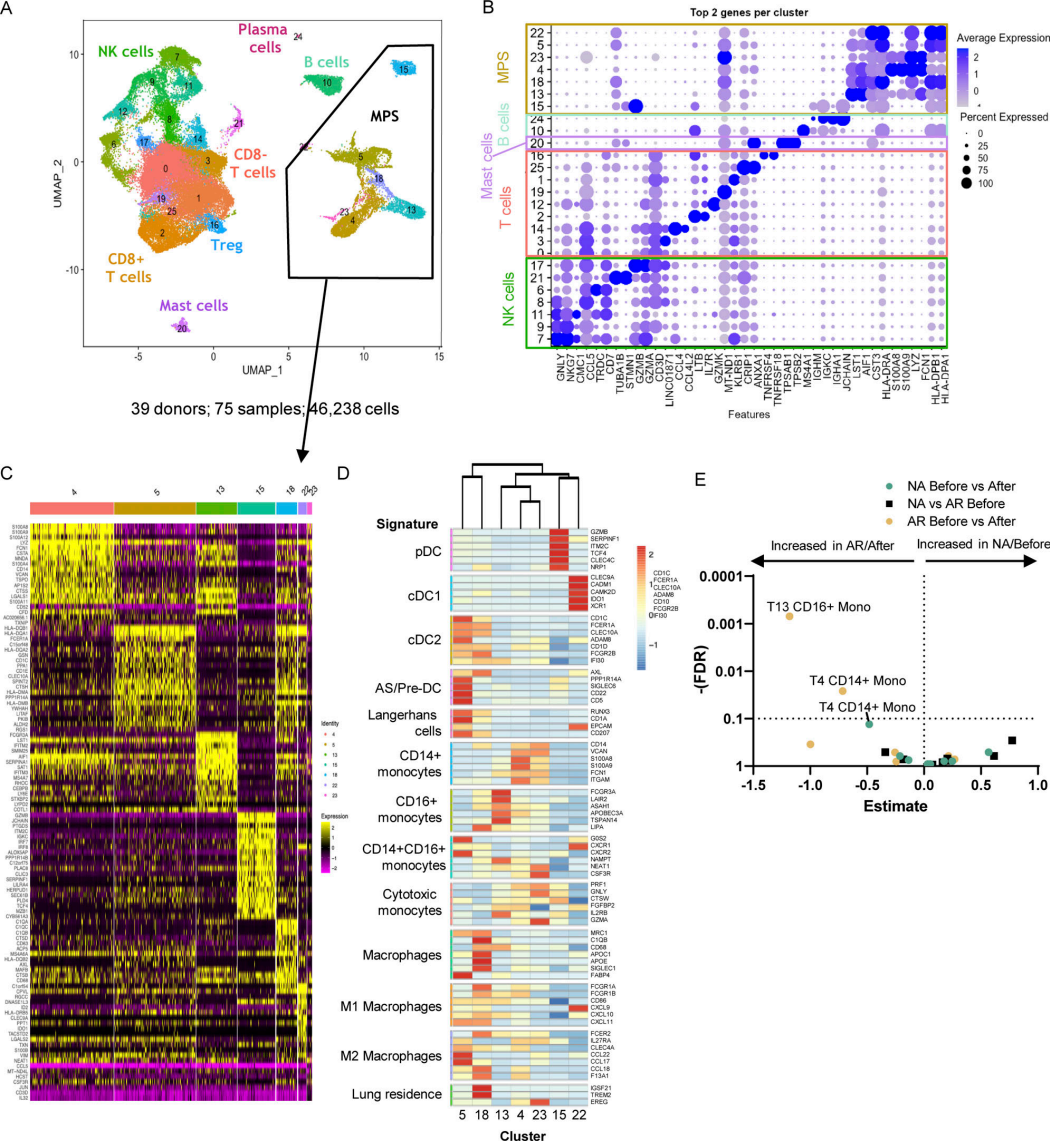
**Nasal biopsy scRNA-Seq results. (A and B)** (A) Annotated UMAP of scRNA-Seq data with (B) top two genes per cluster. **(C)** Heatmap of DEGs between the different MPS clusters. **(D)** Heatmap of average expression for each MPS cluster (identity listed below) of signature genes (listed on the right) of known cell types (left). **(E)** Volcano plot of cluster frequencies between different study groups as determined by GLMM with FDR correction on samples obtained from 18 non-allergic controls and 21 AR patients analyzed in five runs (also see Fig. S5 A; and Tables S7, S8, and S9).

Nasal biopsies were enzymatically treated to yield single cells and either freshly processed or cryopreserved for the analysis of proteomic profiles from CD45^+^ live cells by CyTOF or gene expression by scRNA-Seq, respectively. In the first level of clustering of the CyTOF data, 752,348 cells from 53 samples were divided into 13 lineages (Fig. 1 B and Fig. S1) and identified as CD8^+^ T cells, CD8^+^ mucosal-associated invariant T cells, CD8^−^ T cells (including CD4^+^ T cells), TCRγδ cells, B cells, innate lymphoid cells (ILC) including natural killer (NK) cells, neutrophils, eosinophils, basophils, mast cells, monocytes, MF/DC, and an unidentified group of cells lacking clear cell lineage markers. The scRNA-Seq data consisted of 46,238 cells from 75 samples, yielding 26 clusters (Fig. 2 A and Fig. S1). Major lineages including CD4^+^ and CD8^+^ T cells, B cells, NK cells, monocytes, DC, and mast cells were identified on established markers and enrichment analysis, corresponding with populations detected in the mass-cytometry data using known lineage markers (Table S1). Granulocyte populations, including neutrophils, eosinophils, and basophils were not identified. Cryopreservation will have contributed to the loss of granulocytes, as well as difficulty in their capture with the 10x Genomics Chromium platform (http://10xgenomics.com).

In allergic subjects, several immunological indications of type 2 responses were found by CyTOF (Fig. 1, D and E): influx of eosinophils in response to allergen challenge (Fig. 1, C and D) and higher percentages of pro-inflammatory TH2A CD4^+^ T cells and ILC2 after allergen challenge (Fig. 1 F). Interestingly, basophil percentages were upregulated in all subjects following allergen challenge. These data clearly show that allergic subjects develop a type 2 signature following allergen challenge, which was distinct from non-allergic subjects. Next to granulocytes, clusters of myeloid cells (monocytes and DC/MF) were significantly changed in AR patients in response to the allergen challenge (Fig. 1 D).

### Composition of MPS cells in nasal tissue

Since mucosal MPS cells play key roles in maintaining immune homeostasis or driving inflammation, we further explored phenotype and responses of different MPS clusters to allergen challenge in both groups. Of note, a detailed analysis of lymphoid cell subsets lies outside of the scope of this paper and will be published elsewhere.

**Figure S2.**
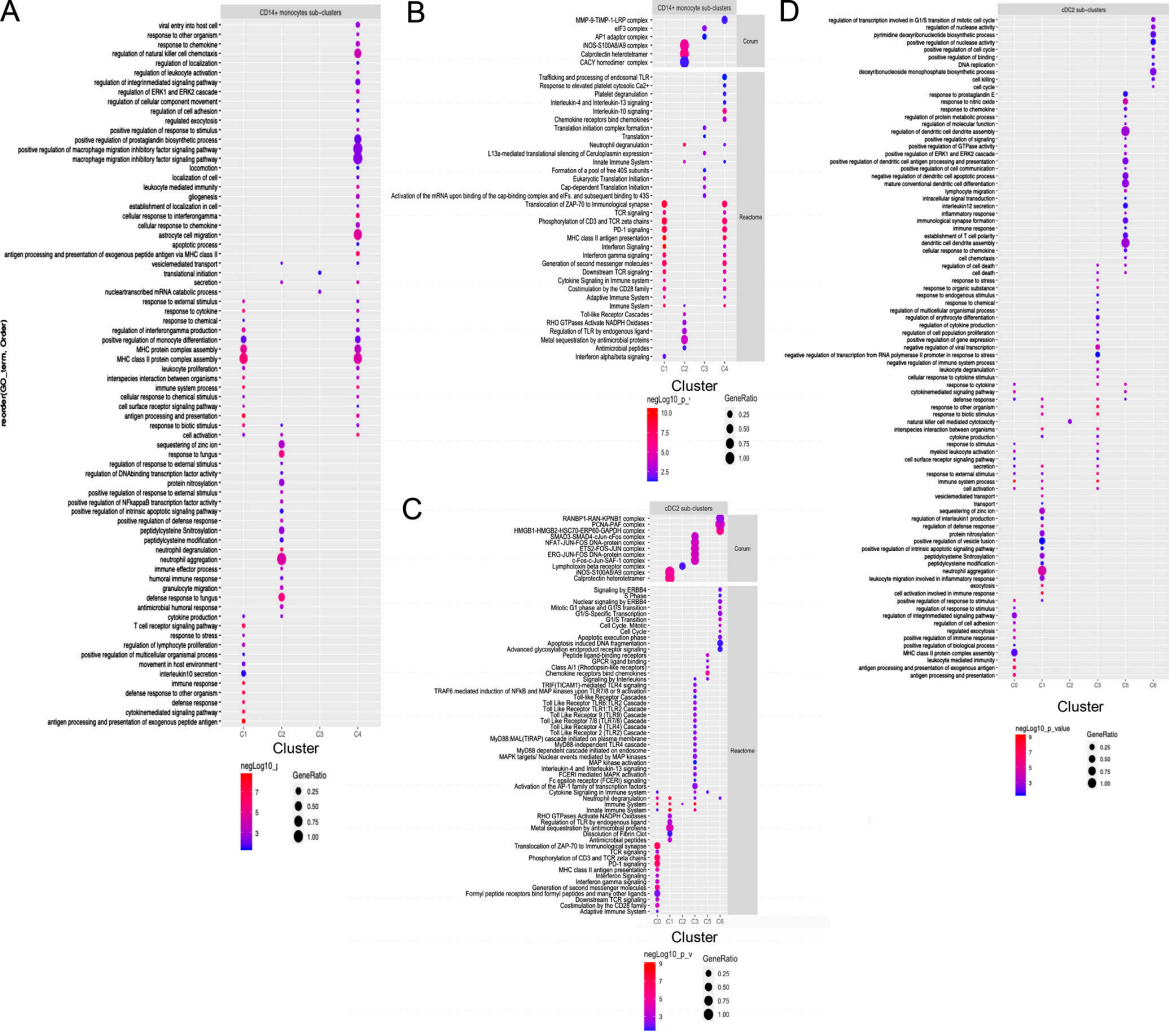
**GO terms corresponding with CD14**^**+**^ **monocyte and cDC2 subcluster gene expression. (A and B)** Dot plot of GO terms of biological processes (GO:BP) (A) and Reactome/Corum (B) generated with the top 40 (as sorted by absolute log_2_ fold change) significantly DEGs (adjusted P value <0.05) between CD14^+^ monocyte subclusters in g:Profiler, and simplified with Revigo (GO:BP only). **(C and D)** Dot plot of GO:BP terms (C) and Reactome/Corum (D) for cDC2 subclusters. Color of dots represent the negative Log_10_ of the adjusted P value and the size represents the gene ratio, equaling the number of DEGs against the number of genes associated with the GO term.

In the scRNA-Seq data, MPS cells were distinguished from other populations based on expression of signature markers *LYZ*, *CST3*, *AIF1*, *LST1*, and *HLA-DRA* (Fig. 2 A and Fig. S1). Top differentially expressed genes (DEGs) of individual MPS clusters were identified (Table S2), and each cluster was annotated based on expression of established markers (Fig. S1) and Enrichr gene set enrichment analysis with the Human Gene Atlas and ARCHS4 Tissues databases (Fig. S2; Karlsson et al., 2021). To support cluster annotation, average gene expression of each cluster was compared with signature profiles of MPS cells (Fig. 2 D) from previous scRNA-Seq studies of peripheral blood and tissue DC, MF, and monocytes (Beyer et al., 2012; Collin and Bigley, 2018; Mitsi et al., 2018; Reyfman et al., 2019; Vieira Braga et al., 2019; Villani et al., 2017). Clusters 4, 13, and 23 display transcript profiles (*S100A8*, *S100A9*, *FCN1*, and *MNDA*) consistent with monocytes. Cluster 4 corresponds with CD14^+^ monocytes, cluster 13 with CD16^+^ monocytes, and cluster 23 with previously described monocytes with a cytotoxic profile (Villani et al., 2017; Fig. 2 D and Table S2). Using generalized linear mixed effect models on the frequencies of the different MPS clusters, we observed significant differences in CD14^+^ monocytes (cluster 4) and CD16^+^ monocytes (cluster 13) before versus after allergen challenge in AR patients (Fig. 2 E).

We also identified several DC clusters: *CLEC10A*^+^, *CD1C*^+^ cDC2 (cluster 5), *CLEC4C*^+^ plasmacytoid DC (pDC; cluster 15), and CLEC9A^+^ cDC1 (cluster 22) populations. Cluster 5 gene expression profile also correlated with several other DC cell types, including Langerhans cells and AS or pre-DC (See et al., 2017; Villani et al., 2017; Fig. 2 C and Table S2). Cluster 18 was identified as MF expressing *APOC1*, *APOE*, and *MRC1*, along with components of both M1 and M2 profiles (Fig. 2 D), previously described in alveolar MF (Mitsi et al., 2018).

### Differential recruitment of monocyte populations in response to allergen challenge

**Figure 3.**
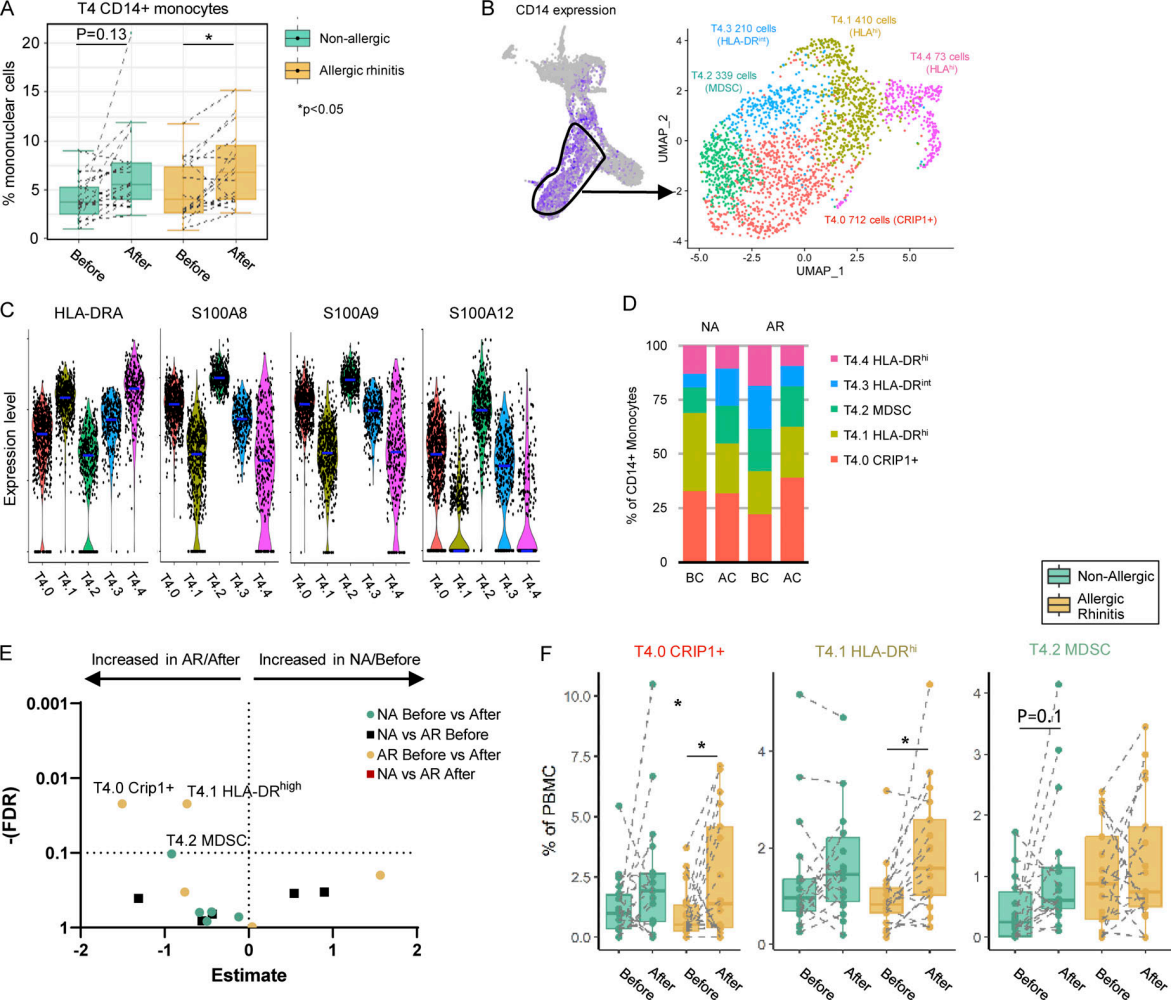
**Subclustering monocyte populations. (A)** Box plot of frequency of CD14^+^ monocytes (cluster T4) in allergic (orange boxes) and non-allergic individuals (green boxes) before and after allergen challenge in the transcriptomic data. *P < 0.05. **(B)** CD14^+^ monocytes (T4) were subclustered, revealing five subclusters. Total cell number and distinguishing marker per subcluster depicted in the plot. **(C)** Violin plot of HLA-DR and S100 protein expression by different CD14^+^ monocyte subclusters. **(D)** Percentage of CD14^+^ monocytes subclusters by study group. **(E)** Volcano plot of cluster frequencies between different study groups/conditions. **(F)** Boxplots of cluster frequencies for clusters showing significant differences. *P < 0.05 as determined by GLMM with FDR correction on samples obtained from 18 non-allergic controls and 21 AR patients analyzed in five runs (also see Fig. S5 A; and Table S7, S8, and S9).

**Figure S3.**
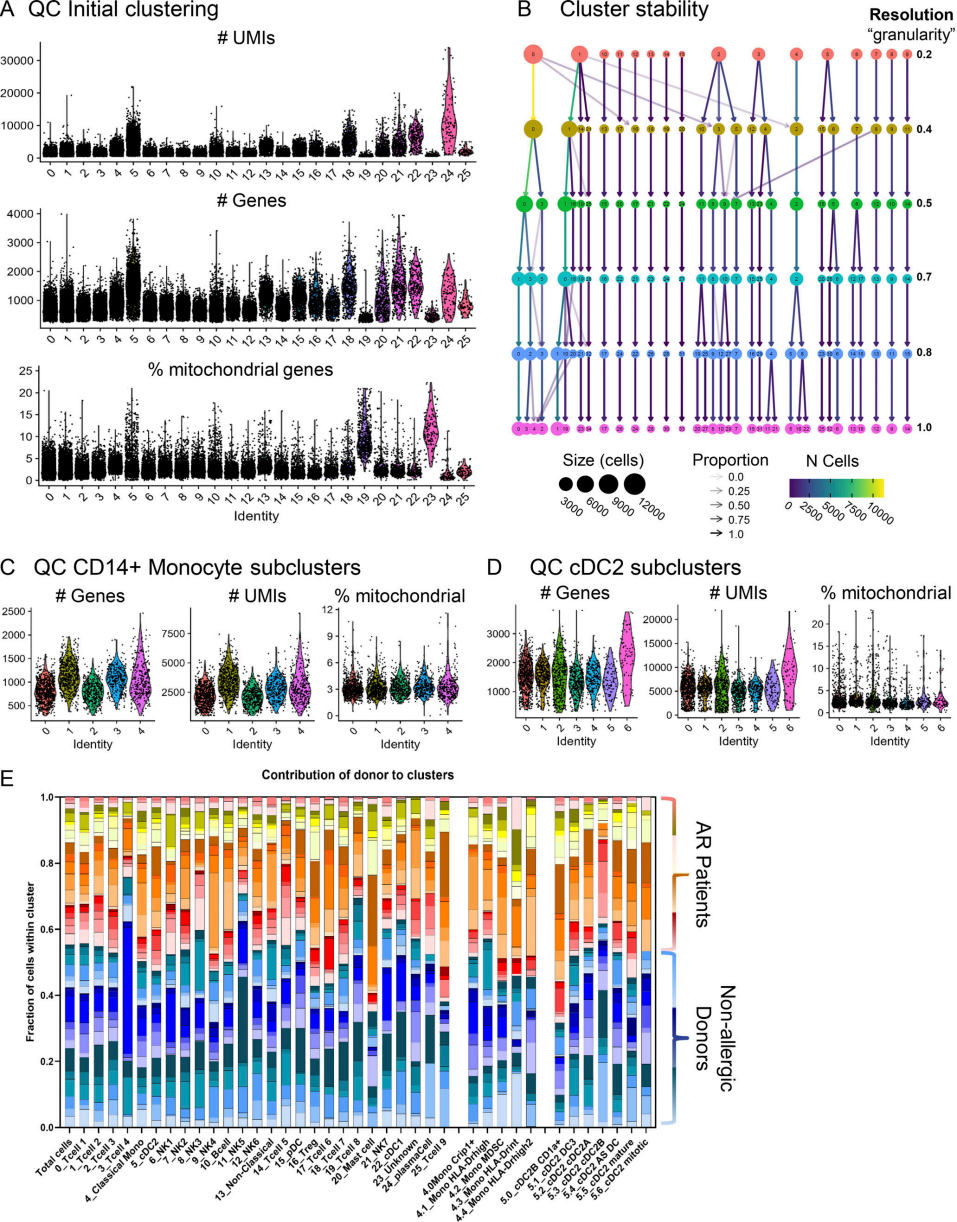
**QC of transcriptomic dataset. (A)** Parameters used for QC of transcriptomic clusters. **(B)** Cluster stability analysis for different values of granularity parameter (“resolution”) used in Phenogrpah clustering. 0.5 was used for clustering in the manuscript. **(C and D)** Parameters used for QC of transcriptomic monocyte (C) and cDC2 (D) subclusters. **(E)** Contribution of each donor to each cluster shows that no donor-/condition-specific clusters exist.

Since percentages of CD14^+^ monocytes increased significantly in both non-allergic and allergic subjects following allergen challenge in the transcriptomic (T) data (Fig. 3 A), we further clustered CD14^+^ monocytes (cluster T4) resulting in five subclusters (Fig. 3 B and Table S3). These monocyte clusters showed no differences in quality control (QC) criteria (Fig. S3) but could be distinguished by HLA-DR and S100 protein expression (Fig. 3 C): two clusters with high HLA-DR expression (T4.1 and T4.4) and three clusters with a low or intermediate HLA-DR expression (T4.0, T4.2, and T4.3). HLA-DR^hi^ monocyte subclusters T4.1 and T4.4 also expressed higher levels of HLA-related and IFN-related genes, important for antigen presentation, cell activation, and response to stimuli (Fig. S2), suggesting a pro-inflammatory role. Furthermore, monocyte subcluster T4.4 expressed genes related to cell adhesion, migration, proliferation, regulation of ERK1/2 cascade, and Th2 cytokine signaling (Fig. S2 and Table S3) along with genes of MF profiles (*MRC1*, *APOC1*). The HLA-DR^low^ monocyte subclusters T4.0, T4.2, and T4.3 expressed higher levels of antimicrobial response genes, such as S100A8 and S100A9 (Fig. 3 C). S100A9 is also associated with immunoregulatory functions in monocyte-derived MF (mo-MF) involving ROS and IL-10 production (Yang et al., 2018). Monocyte subcluster T4.0 expressed higher levels of *CRIP1* (cysteine-rich intestinal protein; Kadrmas and Beckerle, 2004), a molecule with a putative “pro-allergic” role (Cousins and Lanningham-Foster, 2000; Lanningham-Foster et al., 2002). Monocyte subcluster T4.2 expressed the lowest levels of HLA-DR along with the highest levels of *S100A8*, *S100A9*, and *S100A12* (Fig. 3 C) and is comparable with myeloid-derived suppressor cells (MDSC; Zhao et al., 2012).

The cell frequency in the CRIP^+^ monocyte subcluster T4.0 and the HLA-DR^hi^ monocyte subcluster T4.1 showed a significant increase in allergic subjects, whereas the cell frequency of the HLA-DRA^low^ monocyte subcluster T4.2 (resembling MDSC) increased in non-allergic subjects after challenge (Fig. 3, E and F). Of note, in allergic subjects, the subcluster T4.2 was abundantly present at baseline but showed no significant change in frequency change upon challenge. Lastly, the proportions of each subcluster within the CD14^+^ monocytes (T4) change after challenge in non-allergic subjects: the HLA-DR^low^ non-inflammatory subclusters T4.2 and T4.3 form a larger portion of CD14^+^ monocytes, resulting in relatively fewer HLA-DR^hi^ pro-inflammatory monocytes subclusters T4.1 and T4.4 (Fig. 3 D). Indeed, *ISG15* and *IFITM3* gene expression, top gene markers of the HLA-DR^high^ pro-inflammatory monocyte subclusters T4.1 and T4.4, were significantly decreased in CD14^+^ monocytes (T4) of non-allergic subjects following challenge (Table S4).

Combined, we were able to identify five different subclusters of monocytes in the nasal mucosa that show dynamic changes: following allergen challenge in allergic subjects a predominant increase in pro-inflammatory HLA-DR^hi^ CD14^+^ monocyte (T4.0 and T4.1) and CD16^+^ monocyte (T13) clusters, while in non-allergic subjects, primarily, the frequency of HLA-DR^low^ CD14^+^ monocytes (T4.2), MDSC-like cells with anti-microbial/regulatory genes, increased after allergen challenge.

### Difference in effector and anti-inflammatory gene expression of cDC2 in allergic and non-allergic subjects

**Figure 4.**
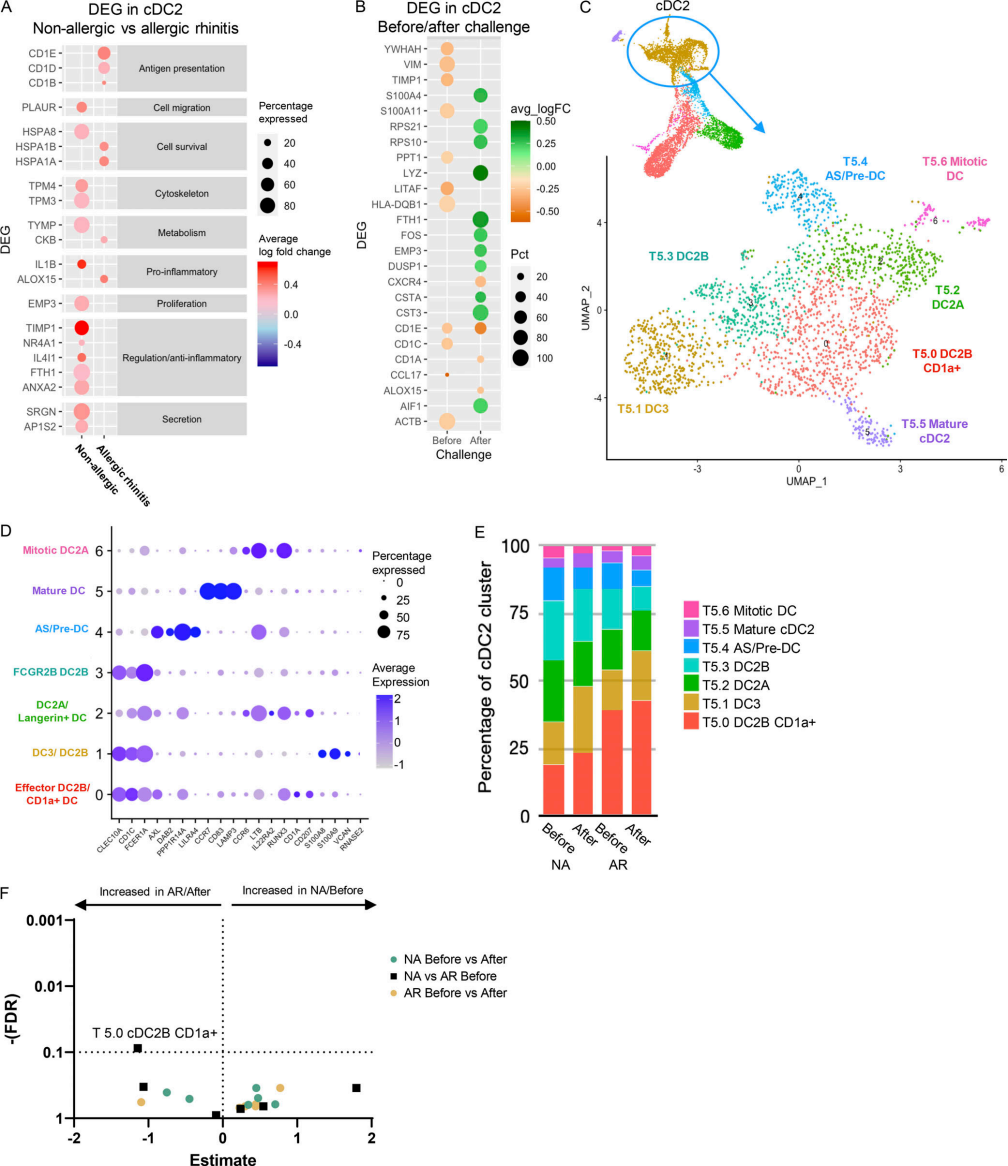
**cDC2 transcriptomic analysis. (A)** Dot plot of DEGs in cDC2 cells (Cluster T5) between before and after allergen challenge for allergic (AR) and non-allergic (NA) individuals. Color intensity represents log fold change (with 0.2 cutoff). **(B)** Dot plot of DEGs in cDC2 cells (Cluster T5) between allergic (AR) and non-allergic (NA) individuals, before and after allergen challenge. Orange represents gene expression higher in allergic individuals, green represents gene expression higher in non-allergic individuals, and color intensity represents log-fold change. Size of the dot represents the percentage of cells in which the genes are expressed. **(C)** Subclustering cDC2 cluster T5 revealed seven subclusters. Total cell number and distinguishing marker per subcluster are depicted in the plot. **(D)** Dot plot of average expression and percentage of cells expressing marker genes of established cell types. **(E)** Percentage of cDC2 subclusters by study group. **(F)** Volcano plot of cluster frequencies between different study groups. P values were obtained by GLMM with FDR correction as determined by GLMM with FDR correction samples obtained from 18 NA controls and 21 AR patients analyzed in five runs (also see Fig. S5 A; and Tables S7, S8, and S9).

Depending on environmental cues, cDC2 can drive both inflammatory and tolerogenic immune responses. Evaluating differential gene expression analysis in cDC2 (cluster T5) showed upregulation of *ALOX15*, *CD1B*, *CD1E*, and *CKB* in allergic subjects following allergen challenge (Fig. 4, A and B; and Table S4). ALOX15 is induced by Th2 cytokines IL-4 and IL-13, of which increased levels were found in nasal secretions following allergen challenge in allergic subjects (Fig. 1 B). CD1 genes belong to the lipid antigen presentation pathway and are associated with DC maturation and increased antigen presentation capacity to T cells following allergen encounter. CKB is primarily expressed in non-classical CD16^+^ monocytes and might indicate the differentiation of these cells into (mo-)DC (Wacleche et al., 2018), following infiltration into the nasal tissue. Interestingly, in non-allergic subjects genes with anti-inflammatory or regulatory functions, or which are expressed in tolerogenic DC (*NR4A1*, *IL4I1*, *FTH1*, *ANXA2*, *TIMP1*), were upregulated (Francesca et al., 2017; Romagnani, 2016; Sands et al., 2009; Tel-Karthaus et al., 2018; Torres-Aguilar et al., 2010; Yue et al., 2015; Zhang et al., 2015). Furthermore, genes related to secretion, cell metabolism, cell survival, and mediator secretion were upregulated in response to allergen (Fig. 4, A and B)*.*

**Figure S4.**
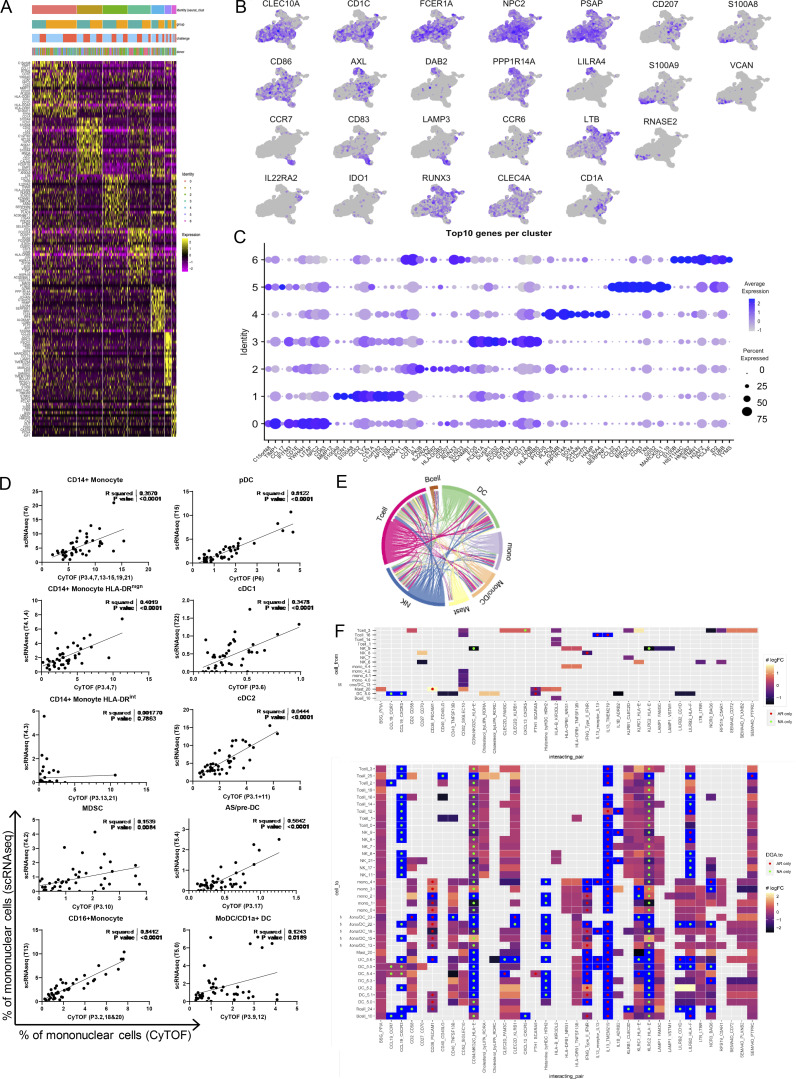
**cDC2 annotation, correlations between CyTOF and scRNA-Seq data, and potential receptor/ligand interactions between clusters. (A)** Heatmap of DEGs between cDC2 subclusters. **(B)** Featureplots of signature genes. **(C)** Top 10 genes per subcluster. **(D)** Correlation plots for cluster frequencies determined by CyTOF and scRNA-Seq. **(E)** Circle plot showing potential ligand–receptor interactions between different cell types and/or within the same cell type (in case multiple clusters of the same type were found) obtained by CellPhoneDB. Width and direction of arrows indicate the number and direction of unique interactions from/to certain cell types, respectively. **(F)** Heatmaps illustrating the results of the CellPhoneDB analysis between non-allergic (NA) controls and AR patients after challenge. Top heatmaps show potential interactions with a DEG for the cluster in which it is differentially expressed, indicated as the first gene on the x-axis. Bottom heatmap shows all of the clusters with which this significantly different gene can have a potential interaction (that does not need to be significantly differently expressed between groups). Colors indicate the log-fold change from non-allergic controls to AR patients, with a blue square indicating that the respective gene is only being expressed in either group. Green and red circles indicate that interaction was only predicted in either non-allergic controls or AR patients, respectively.

Recently, Brown et al. (2019) identified effector/inflammatory cDC2 termed cDC2B and anti-inflammatory cDC2 termed cDC2A. To investigate whether the differences observed in gene expression between the groups upon allergen challenge were related to differences in cDC2 subsets, we further subclustered the cDC2 cluster T5. This resulted in seven distinct subclusters (Fig. 4 C) that were aligned with recently identified cell types (Fig. 4 D) and further characterized by top DEGs of each subcluster (Fig. S4). Five cDC2 subclusters (T5.0, T5.1, T5.2, T5.3, and T5.6) were identified as cDC2 cells based on *CD1C*, *FCER1A*, and/or *CLEC10A*, while the subclusters T5.4 and T5.5 were lacking these markers (Fig. S4 B).

Subcluster T5.2 and T5.6 correspond to antiinflammatory cDC2A based on *CD1C*, *CCR6*, *LTB*, *RUNX3*, and *IL22RA2* expression and absence/reduced expression of CLEC10A, PSAP, and CD86 (Fig. 4 D and Fig. S4; Brown et al., 2019). However, some typical cDC2A markers were absent (CLEC4A, AREG, and NR4A3). Subcluster T5.6 also expressed genes related to mitosis (Fig. S4 C), representing a “mitotic cDC2A” subset (Fig. 4, E and F). The remaining cDC2 subclusters T5.0, T5.1, and T5.3 expressed inflammatory cDC2B markers (*CLEC10A*, *CD1C*, and *FCER1A*), but each expressed a distinct profile (Fig. 4 B). Subcluster T5.0 was distinguished by effector DC markers (*NMES1*, *IFITM3*, and *NPC2*; Brown et al., 2019; Zimmer et al., 2012), CD1a expression, and genes related to cell activation, defense response, and antigen processing and presentation (Fig. S4 and Table S5). In the lung, CD1a^+^ DC are considered potent T cell stimulators (Desch et al., 2016; Masten et al., 2006; van Haarst et al., 1996). Indeed, allergic subjects had a trend toward higher percentages of these CD1a^+^ cDC2 cells (cluster T5.0) compared with non-allergic subjects at baseline (Fig. 4, E and F). Subcluster T5.1 expressed monocyte-like genes (FCN1, SELL, S100A8, and S100A9), corresponding to mo-DC DC3 (Bourdely et al., 2020; Dutertre et al., 2019; Villani et al., 2017). Subcluster T5.3 expressed higher levels of regulatory/inhibitory genes (*FCER1A*, *FTL*, *FCGR2B*, *RGS2*, and *VSIG4*; George et al., 2017; Gueguen et al., 2016; Guilliams et al., 2018; Munawara et al., 2019; Platzer et al., 2015; van Montfoort et al., 2012; Xu et al., 2010) along with MAPK signaling genes (*FOS*, *FOSB*, *JUN*, *JUNB*, and *DUSP1*; Fig. 4 B, Fig. S4, and Table S5), representing the “non-inflammatory” *FCGR2B* cDC2 (Villani et al., 2017; Fig. S4).

The remaining subcluster T5.4 was identified as *AXL *and *SIGLEC 6* positive (AS)-DC or pre-DC (See et al., 2017; Villani et al., 2017), based on *AXL*, *DAB2*, *PPP1R14A*, and *LILRA4* expression (Fig. 4 B) and was increased in allergic patients following allergen challenge (Fig. 4, E and F). Subcluster T5.5 was identified as a small mature DC population (106 cells; Fig. 4, C and D; Fig. S4; and Table S5), expressing *CCR7*, *CD83*, *IL7R*, and *LAMP3*, resembling a *CCR7* and *IL7R *(CD127) expressing DC subset (Fergusson et al., 2019). Genes related to immune regulation and Treg induction (*SAMSM1*, *IL4I1*, *RELB*, *IDO1*, and *IL7R*) were among the top markers for T5.5 (Fig. 4 B).

In conclusion, when analyzing the total cDC2 cluster (T5), the cDC2 in non-allergic controls shows a stronger anti-inflammatory phenotype compared with cDC2 in AR patients. When subclustering the cDC2 cluster (T5), up to five distinct cDC2 subclusters were identified in nasal tissue, of which several have been described by others, including the anti- and pro-inflammatory cDC2A and cDC2B subsets, respectively. A predominance of effector CD1a^+^ cDC2 cells (cluster T5.0) was found in AR subjects who showed a highly pro-inflammatory response after further stimulation. Besides this cluster, no significant differences were found between groups or after challenge, but anti-inflammatory/regulatory gene expression in cDC2 clusters was further increased after challenge in non-allergic individuals.

### Multi-omics integration analysis shows distinct clusters of regulatory and inflammatory myeloid cells

**Figure 5.**
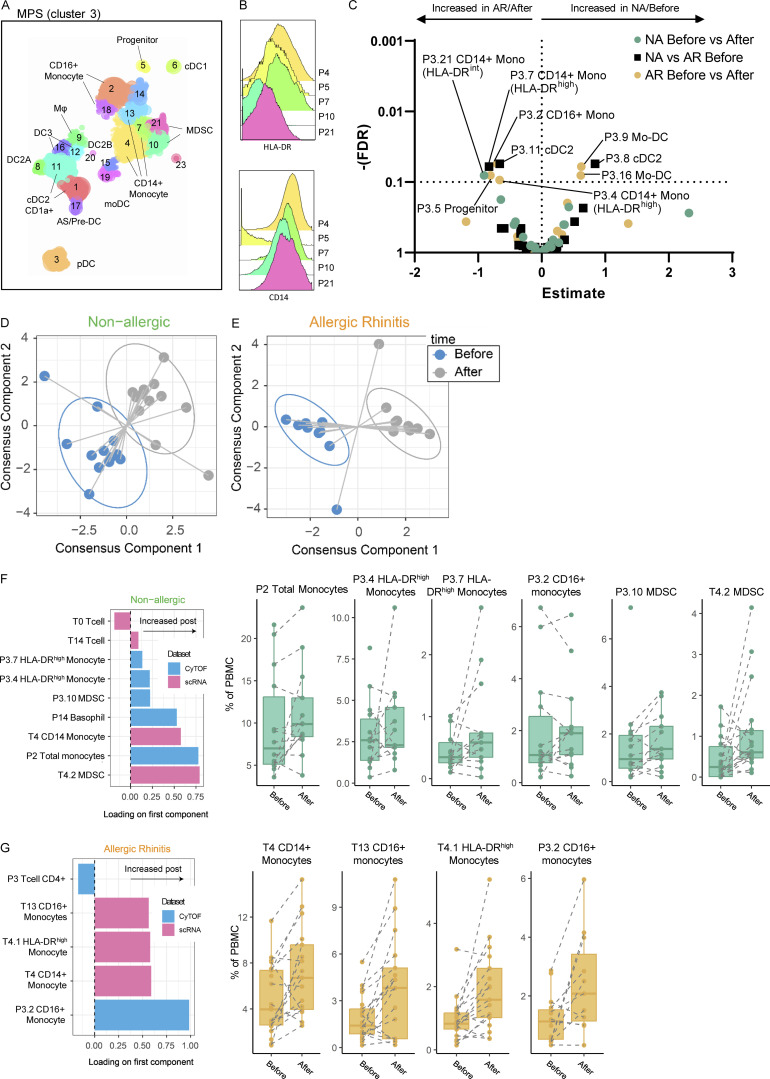
**Subclustering proteomic data and integration of proteomic and transcriptomic data. (A)** Subclustering of MPS (P3; monocytes and P4; DC/MP) cells in proteomic data. **(B)** HLA-DR and CD14 expression of subclusters P4, P7 (both CD14^+^ Monocyte), P5 (progenitor), P10, and P21 (both MDSC). **(C)** Volcano plot of cluster frequencies between different study groups/conditions. **(D and E)** Integration of cluster frequencies as determined from transcriptomic and proteomic data using MixOmics allowed separation of samples obtained before/after challenge for both non-allergic controls and AR patients. **(F)** Loadings of the first component separating non-allergic control samples before/after challenge show which cell (subclusters) allow differentiation between time points with boxplots of the respective (sub)clusters. **(G)** Loadings of the first component separating AR patient samples before/after the challenge show which cell (subclusters) allow differentiation between time points with boxplots of the respective (sub)clusters. P values as determined by GLMM with FDR correction on 14 non-allergic controls and 14 AR patients analyzed fresh (A–C) or on 12 non-allergic controls and 12 AR patients (D–G) analyzed in 5 runs (also see Fig. S5 A; and Tables S7, S8, S9, and S10).

To allow integration of the proteomic and transcriptomic datasets, we reclustered the CyTOF MPS clusters previously identified as HLA-DR^+^CD19^−^CD3 (clusters P3, monocytes and P4, DC and MF; Fig. 1 B and Fig. 5 A). Subclustering resulted in 24 subclusters (P3.1–3.24); identifying populations of CD14^+^ (P3.4, P3.7, and P3.13), CD16^+^ (P3.2 and P3.18), and MDSC monocytes (P3.10 and P3.21); CD34^+^ progenitor cells (P3.5), CD206^+^ MF (P3.9), CD141^+^ cDC1s (P3.6), CD1A^+^ cDC2s (P3.1 and P3.11), cDC2A (P3.8), cDC2B (P3.22), mo-DC (3.19), DC3 (P3.12 and P3.16), AS/pre-DC (P3.17), and CD123^+^ pDCs (P3.3; Fig. 5 A). At baseline, allergic individuals had a higher proportion of cDC2 cluster P3.11 and CD14^+^ HLA-DR^high^ monocyte cluster P3.7, while non-allergics had more non-inflammatory FcεRI^low^ CCR6^+^ HLA-DR^low^ cDC2A (P3.8; Fig. 5, A–C). In allergic subjects, HLA-DR^hi^ monocytes (P3.4), CD16^+^ monocytes (P3.2), and the progenitor cluster (P3.5) increased, while mo-DC clusters (P3.9 and P3.16) and MF (P3.9) decreased following upon allergen provocation (Fig. 5, A–C). In non-allergic individuals, only the MDSC cluster showed a significant decrease after challenge (P3.21; Fig. 5, A–C).

To identify a minimal combination of correlated cell clusters identified in the scRNA-Seq and CyTOF datasets that were associated with the response to challenge, we performed a multi-omics integration analysis on donors that were included in both datasets (11 patients and 12 controls; Fig. 5 D; Singh et al., 2019). This was done separately for the non-allergic and AR groups to see which cell types were most strongly associated with the challenge per group. Based on the leave-person-out cross-validation with tuning of the number of features to select, the first component alone correctly predicted 83.3% of samples in the non-allergic group and 100% of samples in the AR group (data not shown). Addition of the second component did not further improve model accuracies, indicating that the features selected on these first models were sufficient to separate the pre- and post-challenge samples. In the final models, five CyTOF clusters (P2, P3.4, P3.7, P3.10, P14) and four scRNA-Seq clusters (T0, T4, T4.2, T14) were selected in the non-allergic group, while two CyTOF clusters (P3, P3.2) and three scRNA-Seq clusters (T4, T4.1, T13) were included for the AR models (Fig. 5, E and F). Of these 14 features, only one was present in both groups: the scRNA-Seq cluster T4 (CD14^+^ monocytes), which associated with post challenge. For the non-allergic group, monocytes (P2 and T4) and MDSC-like monocytes (P3.10 and T4.2) in both the RNA and CyTOF data were predictive of post challenge. Similarly, although with relative low loadings (<0.25), two clusters of HLA-DR^hi^ monocytes (P3.4 and P3.7) and basophils (P14) in the CyTOF data and one scRNA-Seq T cell cluster (T14) were associated with post challenge, while another scRNA-Seq T cell cluster (T0) was higher with before challenge. In the AR group, in contrast, CD16^+^ monocytes in both the CyTOF (P3.2) and scRNA-Seq (T13) were associated with post challenge. HLA-DR^hi^ monocytes in the RNA data (T4, T4.1) were also associated with post challenge, while total CD4^+^ T cells (P3) in the CyTOF were linked to before challenge (Fig. 5, E and F). The correlation of selected features in each of the two models was strong between datasets (Fig. 5 D). Moreover, the features that were selected in the AR model clustered mostly separately from the features selected in the non-allergic group, suggesting that these cell clusters are not coregulated and are reflecting separate processes in the two groups.

### Receptor–ligand interactions between clusters

Using CellPhoneDB (Efremova et al., 2020), we assessed potential receptor–ligand interactions with cell clusters (Fig. 2, A and B) for genes that differed significantly following allergen challenge for each group (Fig. S4, E and F). Arrow origins indicate in which cluster the significantly differing gene was found (Fig. S4 E). A total of 37 interactions was found, several of which were present only/mostly in cell clusters of non-allergic controls (only: CCL19-CCR7 and CCL19-CXCR3; mostly: CD94:NKG2C-HLA-E, CXCL13-CCL5, and KLRC2-HLA-E) or AR patients (only: IL-13-IL-13R, and IL-13-TMEM219; mostly: CD38-PCAM1, FTH1-SCARA5, and IFNG-IFNGRTII; Fig. S4 F; top heatmap indicates source cell, bottom indicates potential interacting partner cell). Interestingly, the disparate interactions found only in non-allergic controls (related to chemokine interactions) or AR patients (related to type 2 immunity) again point to the activation of different cell systems following allergen challenge in the different groups. However, we should consider that the predominant chemokine response observed in non-allergics is partly caused by a reduction in cell numbers of the cDC2 subcluster T5.0 in allergic patients following allergen challenge.

Overall, the multi-omics integration analysis with regards to the MPS cells particularly points toward a role for different monocyte clusters, discrimination between AR and non-allergic individuals in their response to allergen challenge. Possibly, the differences in cDC2 subsets are more related to a differential gene expression per subset, rather than differences in cell numbers/percentages.

### Stimulation and cytokine assessment of nasal tissue MPS cells

**Figure 6.**
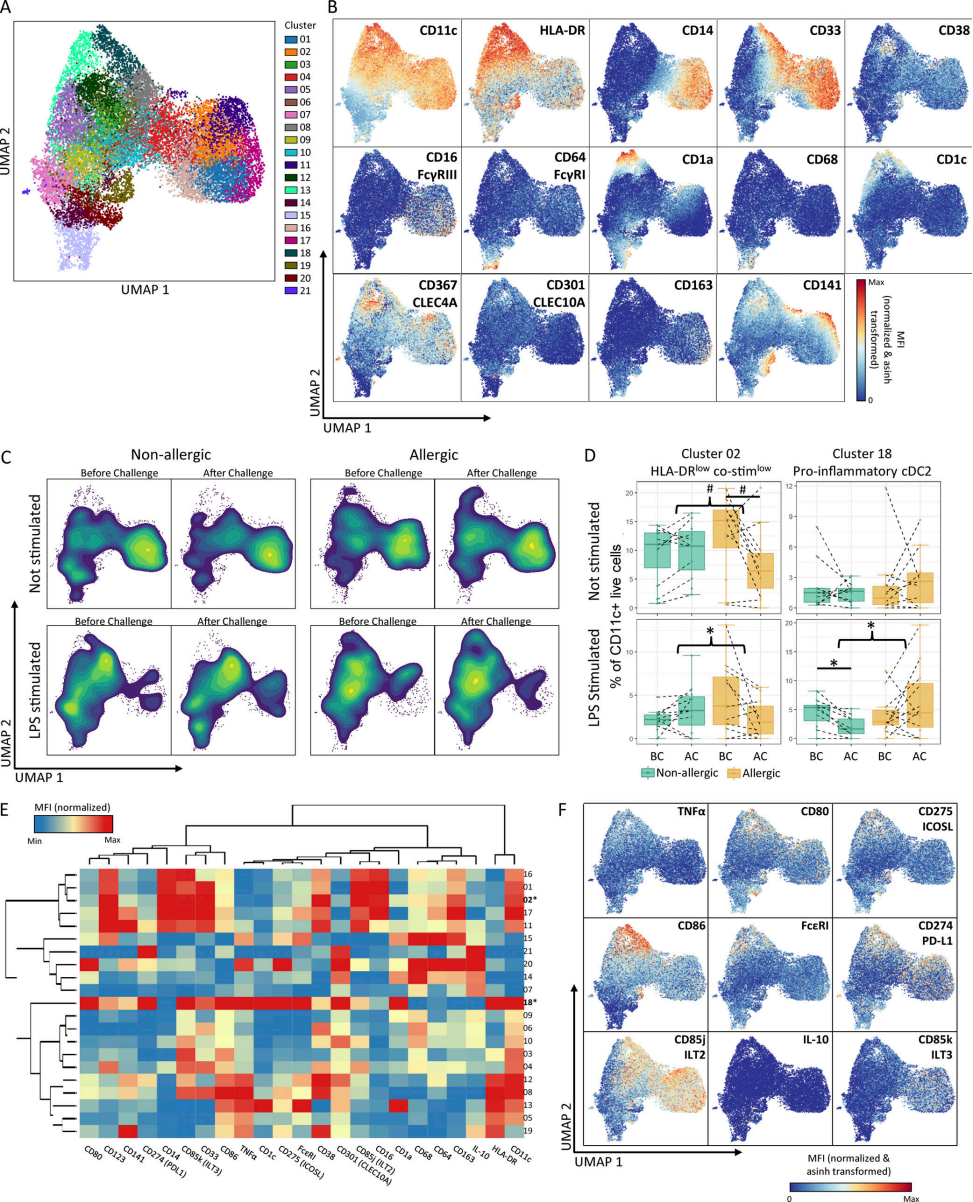
**Ex vivo stimulation of nasal biopsy MPS cells. (A)** UMAP of the 21 clusters generated from CD11c^+^ cells after culture of nasal biopsies with or without LPS. **(B)** UMAP from A colored by a marker for each of the markers used for clustering as well as CD16. **(C)** Density plots of UMAP for non-allergic controls and allergic patients before (BC) and after challenge (AC), cultured with or without LPS show clear differences in cell abundance between groups. **(D)** Clusters 2 and 18 showed significant differences between non-allergic controls and allergic individuals or in the response to HDM challenge between the two groups. Lines indicate differences within groups and braces indicate a difference in response between groups as determined by linear mixed-effect model. # indicates P < 0.05 before FDR correction, * indicates P < 0.05 after FDR correction as determined by GLM. **(E)** Heatmap indicating the relative expression of lineage and activation markers in each cluster. Clusters showing significant differences between groups are in bold. **(F)** UMAP of the activation/tolerance markers and cytokines that were not used for clustering. Data represent 45 biopsies obtained from 10 non-allergic controls and 13 AR patients analyzed in three separate experiments.

To assess whether the differences in monocyte and cDC2 subset numbers and transcriptomes corresponded to functional differences, cryopreserved single-cell suspensions obtained from nasal biopsies were thawed and stimulated with LPS or medium for 24 h and analyzed for TNFα and IL-10 secretion combined with expression of cell surface markers for cell lineage, activation, and tolerance (Table S6). Clustering CD11c^+^ cells based on lineage marker expression revealed 21 different clusters (Fig. 6, A and B). Overall IL-10 secretion levels remained very low in all the clusters analyzed, while TNFα secretion showed a large variation between the different subsets. From the 21 clusters, two (clusters 2 and 18) showed significant differences between allergic and non-allergic subjects (Fig. 6, C and D). Cluster 2 comprised HLA-DR low monocytes with low levels of co-stimulatory molecules CD80, CD86, and CD275 and high levels of markers associated with tolerogenic DC CD85j and CD85k, resembling MDSC-like monocytes (cluster T4.2, P3.10, and P3.21) and which produced little TNFα compared with other MPS subsets. In LPS-stimulated samples, the percentage of cells in cluster 2 was increased after allergen challenge in non-allergic individuals, while it was decreased in allergic individuals after allergen challenge compared to before challenge (Fig. 6, E and F). In unstimulated samples, a similar trend toward a decrease in allergic individuals was observed, but this was not significant after false discovery rate (FDR) correction. These results are in line with the increase in MDSC observed by CyTOF and scRNA-Seq (Fig. 3, E and F; and Fig. 5 C). Cluster 18 displayed an opposite response to cluster 2. Cluster 18 consisted of cDC2 expressing inflammatory markers such as CD1a, high levels of HLA-DR and FcεRI, co-stimulatory molecules (CD80, CD86, and CD275), thereby resembling the inflammatory cDC2 clusters (T5.0, T5.1, T5.3 P1, and P11; Fig. 6, E and F). The cells in this cluster released high levels of TNFα, confirming their pro-inflammatory phenotype. Upon LPS stimulation the percentage of cDC2 in this cluster was decreased in biopsies from non-allergic subjects after challenge compared with before challenge, while in allergic subjects the percentage increased after allergen challenge. Again, these data confirm our findings observed in mass cytometry and scRNA-Seq (Figs 3, 4, and 5).

Taken together, these data confirm that allergen challenge in non-allergic subjects induces a non-inflammatory, HLA-DR^low^ monocyte subset that retains its HLA-DR^low^ phenotype after stimulation with LPS, while in allergic subjects an inflammatory cDC2 subset is increased in number that responds to LPS by expression of multiple co-stimulatory molecules and releases TNFα.

## Discussion

Mucosal surfaces in the upper airways are constantly challenged by pathogens and foreign particles. While the innate role of myeloid cells in protecting against invaders is well documented, their role in allergic responses and maintaining immune homeostasis at mucosal surfaces is understudied. We performed a comprehensive single-cell proteome and transcriptome analysis on nasal tissue biopsies, following controlled allergen challenge in allergic and non-allergic individuals, and identified MPS cell clusters that were involved in inflammation and tolerance and which largely segregated in allergic and non-allergic subjects following allergen challenge.

Five subclusters were identified within CD14^+^ monocytes (transcriptomics), which could be grouped into HLA-DR^hi^ and HLA-DR^low^ populations. In non-allergic individuals, only HLA-DR^low^ CD14^+^ monocytes increased in frequency in nasal tissue in response to allergen, whereas in allergic subjects, the frequency of HLA-DR^hi^ CD14^+^ monocytes and CD16^+^ monocytes significantly increased. HLA-DR^high^ CD14^+^ CD16^−^ and CD14^+^ CD16^+^ monocytes are considered pro-inflammatory and their recruitment has been implicated in various inflammatory diseases (Kapellos et al., 2019; Linton et al., 2012). CD16^+^ CD14^−^ monocytes have been termed patrolling monocytes and are recruited to areas of inflammation to assist in tissue repair. They have been shown to differentiate into mo-DCs, with migratory abilities (Randolph et al., 2002) that produce TNFa (Wacleche et al., 2018) or induce higher levels of IL-4 production from CD4^+^ T cells compared with CD16^−^ monocytes (Randolph et al., 2002; Rivas-Carvalho et al., 2004; Sánchez-Torres et al., 2001). Thus, the increased number of HLA-DR^high^ CD14^+−^ and CD16^+^ monocytes in allergic subjects following challenge will perpetuate the Th2 type response.

Interestingly, HLA-DR^low^ CD14^+^ monocyte clusters in non-allergic subjects expressed genes similar to MDSC, including the anti-microbial S100A8/9/12 genes (Ben et al., 2019; Veglia et al., 2021). MDSC are originally known for their anti-inflammatory capabilities and accumulate in cancer and chronic inflammatory conditions (Dorhoi and Du Plessis, 2018; Kolahian et al., 2016; Mengos et al., 2019) as well as in infections such as COVID-19 disease or influenza (Falck-Jones et al., 2021). Adoptive transfer of MDSC or induction of those cells (Arora et al., 2010) has been shown to dampen airway inflammation in a mouse-model for asthma through Arginase-1 and NO synthase-2 (Sendo et al., 2019; Tian et al., 2019; van Geffen et al., 2021). The striking combination of antimicrobial and regulatory genes we observed here in non-allergic individuals has also been found in mo-MF with immunoregulatory functions, expanding in the gut after stimulation by microbial metabolites (Schulthess et al., 2019; Yang et al., 2018). Although elevated levels of MDSC have been reported in allergic diseases like asthma or rhinitis—as we do here as well (Fig. 3)—we do show for the first time an increase in MDSC in non-allergic healthy individuals during allergen exposure, which was experienced without any clinical symptoms. Importantly, the increase corresponded to an elevated cellular non-inflammatory response, underlining an important role for MDSC to maintain immune homeostasis in non-allergic individuals and suggesting an impairment of its function in patients. The exact mechanism determining whether a pro- or anti-inflammatory monocyte response is mounted remains to be determined. The presence of Th2 cells in the tissue of AR patients is a likely candidate (as demonstrated for the airways of patients with allergic asthma; Alladina et al., 2023; Cho et al., 2016), either or not boosted by local chemokines, as those were prominently found among differentially expressed ligand–receptor interaction pairs described in Fig. S4, E and F.

In the cDC2 compartment, we found different cDC2 clusters, of which the effector CD1a^+^ cDC2 subpopulation dominated in allergic subjects at baseline, and these cells increased in number after allergen challenge in allergic individuals. This population seems similar to the cDC2B subset, which has been defined as pro-inflammatory by Brown et al. (2019). Furthermore, previous studies on nasal mucosa have found elevated numbers of mature CD1a^+^ DC in AR compared with non-allergic individuals and an influx of CD1a^+^ cDC2 after allergen challenge (KleinJan et al., 2006; Melum et al., 2014; Reinartz et al., 2011)*.* This supports our findings regarding the CD1a^+^ cDC2 cells identified here. CD1a^+^ cDC2 cells can rapidly activate pre-existing allergen-specific Th cells as HLA genes are upregulated (Table S5). In contrast, in non-allergic individuals, the various cDC2 subpopulations are more balanced and include the anti-inflammatory cDC2A population as well as FCGR2B^+^ cDC2 cells (Brown et al., 2019; Villani et al., 2017). FCGR2B cDC2 represents a separate cluster distinguished by higher expression of several anti-inflammatory/regulatory markers (*FCER1A*, *FCGR2B*, *FTL*, and *VSIG4*), suggesting a regulatory role to maintain immune homeostasis. Expression of MAPK signaling genes (*FOS*, *JUN*) was also increased in FCGR2B cDC2B. MAPK pathways are known to be required for myeloid immune suppression or could suggest that they are responding to inflammatory cytokines or stress (Yu et al., 2021). Furthermore, Villani et al. (2017) made the distinction between “non-inflammatory” FCGR2B^+^ DC2 and “inflammatory” FCGR2B^−^ CD163^+^ CD36^+^ DC3. Although those were analyzed from blood, our tissue populations seem similar, and expression of some signature genes of non-inflammatory cDC2 was enhanced in cDC2 cells from non-allergic individuals following allergen challenge.

A previous study with grass and tree pollen challenge showed a rapid influx of CD14^+^ HLA-DR^+^ monocytes in the nasal tissue of AR subjects. This was detectable 12 h after allergen challenge (Eguíluz-Gracia et al., 2016), followed by recruitment of Th2 cells and eosinophils. Transcriptome analysis of CD45^+^HLA-DR^+^ cells within nasal biopsies after an 8-d allergen challenge revealed upregulation of genes regulated by IL-4 and or IL-13, including *ALOX15*, *CD1A*, and *CD1B*, similar to our findings here following HDM challenge. The origin of these transcripts (monocytes, cDC, or pDC) was not identified in that study due to low cell numbers. We have now determined that these transcripts are derived from CD1A^+^ cDC2 cells (within subcluster T5.0 of cDC2 cells), expressing *CCL17* and other IL-4–inducible genes. In contrast to our findings in the transcriptome data, Eguíluz-Gracia et al. (2016) did not detect an increase in monocytes of non-allergic subjects 12 h after to pollen allergen challenge using flow cytometry*.* This is consistent with total monocytes (P3) from our proteome data, while subclusters (MDSC P3.21 and progenitor cells P3.5) did increase in numbers (Fig. 5, A–C), suggesting that further phenotyping is necessary to visualize physiological and mechanistic processes at the nasal mucosa in non-allergic individuals.

In our study, we analyzed a large number of subjects and samples by both mass cytometry and scRNA-Seq. The use of barcoding has enabled this for scRNA-Seq analysis by greatly reducing the costs per sample. A downside to this method, however, is that fewer cells could be analyzed per sample (an average of 600 cells). Therefore, cell numbers were sometimes too low to contribute to the smaller (sub)clusters. This may have affected the identification of DEGs between groups or conditions as well as prevented differences in cell subset frequencies to reach significance. This limitation was partly compensated by the multi-omics integration analysis of two independent datasets, allowing for more confidence with respect to changes in (sub)clusters despite the low cell number counts. Furthermore, we employed statistical models with weighing to account for differences in cell numbers between samples and which used average frequencies per individual as input. The large number of samples required the samples to be processed on multiple lanes of the 10× chromium system. This could lead to batch effects, but these could be mitigated using Seurats’ SC Transform (SCT). This resulted in clustering without batch or patient-specific cell clusters (Fig. S3 E). Furthermore, the statistical methods employed would not allow batch or patient-specific clusters to reach statistical significance.

Another potential weakness in our study is the use of Derp1 obtained from HDM extract. These extracts are known to contain LPS, which has been shown to induce neutrophil, CD14^+^ CD16^−^ monocyte, and CD1c^+^ DC recruitment to the airways in non-allergic individuals. Additionally, monocytes expressed lower levels of antigen-presenting genes and higher levels of LPS-response genes (e.g., *IL1A*, *IL1B*), and retained *S100A8/9* and *SELL* expression in comparison to steady state, which could be similar to infiltrating HLA-DR^low^ monocytes/MDSC we also observed (Braga et al., 2004; Jardine et al., 2019; Kvarnhammar et al., 2012). The concentration of LPS in our extract was only 1.49 pg/ml. Although an absence of response to such a dose can only be determined by another nasal challenge, the LPS dose was orders of magnitude lower than that used to induce nasal inflammation (>60 μg; Braga et al., 2004; Jardine et al., 2019; Kvarnhammar et al., 2012). Furthermore, the absence of a control group with AR, but no Derp1 sensitization, makes it impossible to distinguish and determine which components of the observed immune response are antigen specific. Lastly, biopsies can be contaminated with peripheral blood, especially when bleeding occurs during tissue collection. However, biopsies are washed extensively before tissue digestion, greatly reducing blood contamination. Furthermore, previous experiments from our lab have shown cell frequencies to differ between biopsies and peripheral blood (Jochems et al., 2019), and multiple cell types not present in blood (e.g., mast cells) were observed in our data, as well as reductions in cell types earlier described to migrate from the tissues following allergen challenge, such as specific DC subsets. Taken together, this indicates that most of the cells analyzed originate from mucosal tissue and if any blood contamination was interfering, the chances this may have had a dominant effect in the conclusions drawn are very slim.

Collectively, our data indicate a distinct, local, innate immune response to allergen in non-allergic individuals, characterized by infiltration of non-inflammatory HLA-DR^low^ CD14^+^ monocytes, similar to MDSC and enhanced transcriptional activation of non-inflammatory cDC2, characterized by tolerogenic genes. The response in allergic individuals indicates a role for infiltrating pro-inflammatory HLA-DR^hi^ CD14^+^ monocytes and CD16^+^ monocytes, and *ALOX15* upregulation in CD1A^+^ cDC2, in the development or maintenance of allergic responses. Future therapies should be tailored toward those early innate monocyte populations, either enhancing or reducing their activity for the treatment or (secondary) prevention of allergic airway disease.

## Materials and methods

### Study design and sample collection

Adult subjects sensitized to HDM who fulfilled the Allergic Rhinitis and Its Impact on Asthma criteria (Bousquet et al., 2008) for moderate to severe persistent AR and non-allergic subjects without sensitization to inhalant allergens and without clinical features of AR were included in the study. Allergic subjects had a history of perennial rhinitis symptoms with or without conjunctivitis and were skin prick test positive to HDM extract (Der. Pteronyssinus, ALK-Abello, Denmark; skin wheal area ≥0.4 histamine equivalent prick area, corresponding with the internationally accepted wheal diameter of ≥3 mm). Non-allergic subjects had no history of AR and were skin prick test (SPT) negative to HDM, birch pollen, grass pollen, cat and dog, or other animals with which they were in daily contact with. Exclusion criteria consisted of pregnancy, nasal polyps, and anatomical or other disorders of the nose. In total, 30 allergic subjects and 27 non-allergic subjects were recruited (Table 1).

### Nasal allergen provocation

Nasal allergen provocations were performed using a Der p1 extract (HAL Allergy), which contained 1.49 pg/ml LPS. Provocations were performed according to the protocol shown in Fig. 1 A, as described previously (Boot et al., 2008; de Graaf-in't Veld et al., 1997; Terreehorst et al., 2002). Provocations were performed once a day for three consecutive days. Nasal corticosteroids and antihistamines were withdrawn 3 wk and 3 d, respectively, before the provocation. Provocations were performed in the absence of total nasal obstruction or infection as assessed by rhinoscopy. The nasal provocation on the first day was performed with three increasing doses of allergen extract (100,1000,10,000 BU/ml) into both nostrils at 10-min intervals after sham challenge with PBS containing human serum albumin 0.03% and benzalkonium chloride 0.05%. (HAL Allergy). The nasal response was assessed with a score system according to Lebel (Lebel et al., 1988). The second and third challenges in one nostril were performed with PBS and HDM 10,000 BU/ml only. PBS and the allergen extract were sprayed into the nostrils with a nasal pump spray delivering a fixed dose of 0.125 ml solution. Nasal provocations were performed out of pollen season between September 2017 and February 2018. Non-allergic controls and AR patients were challenged in a randomized manner to prevent bias arising from seasonal changes and differences in storage times.

### Nasal biopsies

Biopsies were taken twice: once before the allergen provocations from one nostril and once 1 d after the third allergen provocation from the other nostril. Three 2-mm^2^ biopsy samples were taken from the inferior turbinate. Beforehand, the nasal mucosa was anesthetized by inserting three pieces of cotton wool on the mucosa soaked in 5% cocaine hydrochloride that was inserted onto the mucosa. 15 min were allowed for the local anesthetic to take effect. Following this, biopsy samples were taken using a specially developed Gerritsma biopsy forceps (Phoenix Surgical Instruments Ltd). Hemostasis was achieved by packing the nose with cotton wool balls soaked in 0.5 ml of 1:1,000 adrenalin. These were removed after 15 min and the nose was then examined for sites of bleeding and, if present, areas were cauterized with a bipolar electrocoagulation (Erbe Surgical Systems). The patient was observed for a further 15 min and cauterization was performed again if necessary.

### Nasal biopsy digestion

At each visit, before and after allergen challenge, three individual nasal biopsies were taken and stored in 5 ml of cold 10% FCS IMDM for no longer than 1 h. The biopsies were then finely cut using a sterile scalpel and digested in 10% FCS IMDM with Liberase TL (125 μg/ml; Sigma-Aldrich) and DNAse I (100 μg/ml; Sigma-Aldrich) overnight at 4°C. Different protocols were tested, but in which overnight 4°C digestion performed optimally equally well for us compared to 1 h at 37°C. Because of logistical reasons, the overnight incubation step was chosen. After digestion, an equal volume of FCS was added and the suspension was vortexed for 30 s. The biopsies were then pressed through a 100-μm filter, rinsed thoroughly with IMDM, and filtered over a 70-μm filter. Cells were spun down for 10 min at 400 *g* followed by red blood cell lysis with an osmotic lysis buffer (eBioscience). Cells were then resuspended in RPMI 1640 and counted. From each sample, 1 × 10^5^ cells were removed and cryopreserved in 10% DMSO in FBS using a Mr. Frosty freezing container. The remaining cells were spun down for 10 min at 400 *g* and resuspended in staining buffer for mass cytometry staining with metal-conjugated antibodies.

### Mass cytometry measurements and analysis

Antibody-metal conjugates (Table S1) were purchased from Fluidigm or conjugated using 100 μg of purified antibody and the Maxpar X8 Antibody Labelling kit (Fluidigm). Conjugated antibodies were stored at 4°C in Antibody Stabilizer PBS (Candor Bioscience, GmbH). All antibodies were titrated prior to use. Cells were stained with metal-conjugated antibodies for mass cytometry according to the Maxpar Surface Staining protocol V2. Briefly, cells were stained with 1 μM intercalator Rh-103 (Fluidigm) for 15 min, washed, and incubated for 10 min with TruStain FcX-receptor block (BioLegend) prior to a 45-min incubation period with the antibody cocktail (Table S1). Cells were washed twice with staining buffer and incubated for 1 h with 1 ml of 1,000× diluted 125 μM Cell-ID intercalator-Ir191/Ir193 (Fluidigm) to stain DNA for cell identification. Cells were then washed three times with staining buffer and twice with deionized H_2_O. Finally, EQ Four Element Calibration Beads were added for normalization, and cells were acquired on a Helios 2 mass cytometer (DVS Sciences). In addition to the metals included in the panel, channels to detect intercalators (103Rh, 193Ir, and 193Ir), calibration beads (140Ce, 151Eu, 153Eu, 165Ho, and 175Lu), and contamination (133Cs, 138Ba, and 206Pb) were included during measuring. After data acquisition, the files were normalized with the reference EQ passport P13H2302 and, where applicable, concatenated. Raw data files are available on the Flow Repository (accession number FR-FCM-Z69M). The median biopsy yield measure by mass cytometry was 6.76 × 10^4^ cells (interquartile range: 2,46 × 10^4^ to 9,72 × 10^4^) per sample, of which 80.6% (median) were stromal cells. Viable, single CD45^+^ cells were pregated according to a previously described gating strategy (Bagwell et al., 2020) and exported as new FCS files with Flowjo V10 for Mac (FlowJo LLC). Data was transformed with hyperbolic arcsine using a cofactor of 5 and distinct cell clusters were identified with Hierarchical Stochastic Neighbor Embedding in Cytosplore (https://www.cytosplore.org/; Fig. S1). Clustering was performed in two levels, once to determine the lineages present and another on the identified lineages. The second level of clustering on the MPS lineage was performed without the FcεRI marker, as inclusion of this marker led to additional clusters solely based on FcεRI expression, which differed significantly between the non-allergic and allergic cohort (Fig. 2 A). Cytosplore output was analyzed with the “Cytofast” package (https://rdrr.io/github/KoenAStam/cytofast; Beyrend et al., 2018) in RStudio (Rstudio, Inc., http://www.rstudio.com), to produce heatmaps, scatterplots of subset abundance, and histograms of the median signal intensity distribution of markers. Cell numbers were normalized to the total number of CD45^+^ cells or mononuclear cells (CD45^+^ cells with granulocyte numbers removed to compare with findings from the transcriptomic data) for each subject to correct for varying biopsy yields.

### scRNA-Seq

**Figure S5.**
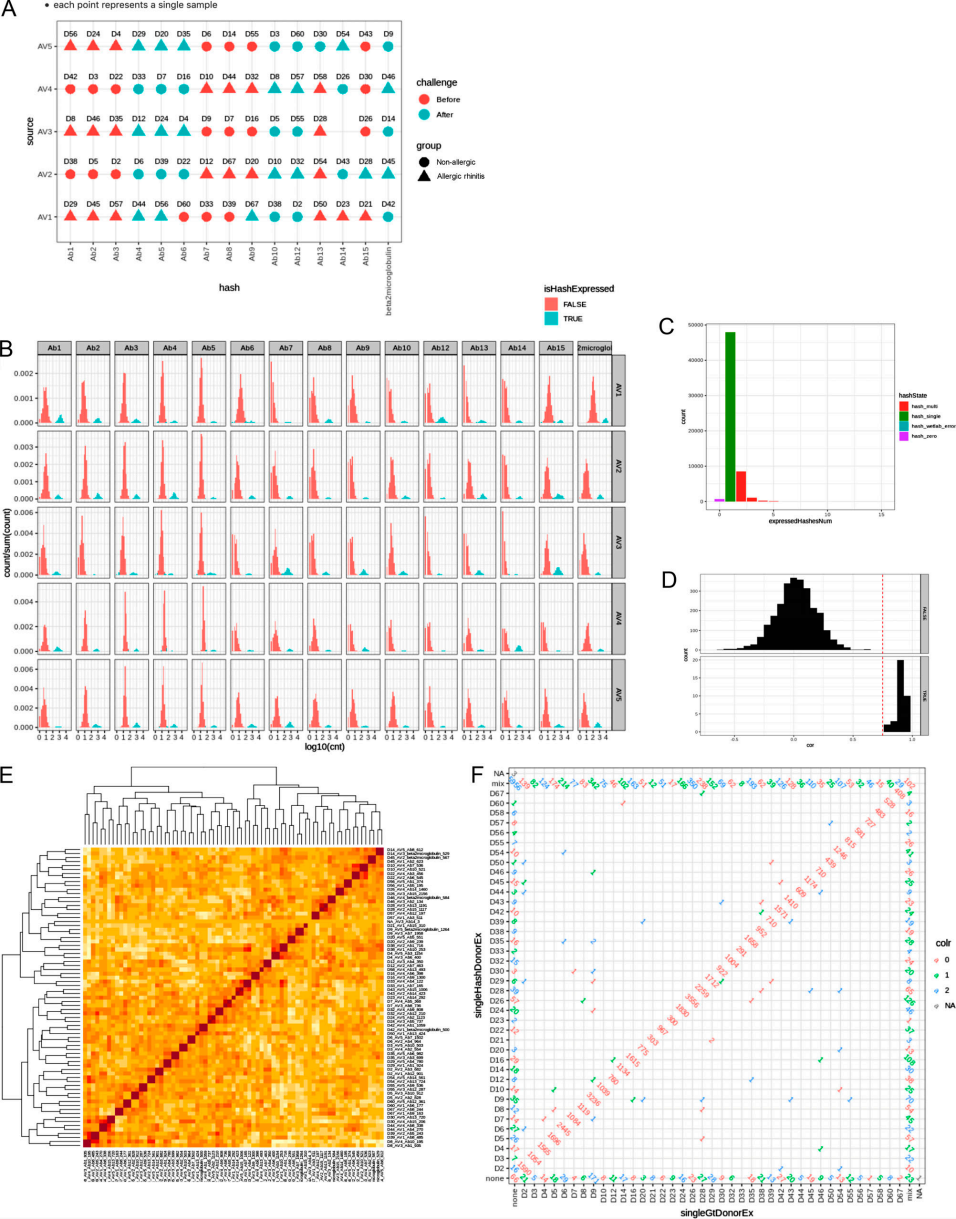
**Transcriptomic data debarcoding. (A)** 15 samples were pooled for each run on the 10× chromium encapsulation chip. Samples were assigned to a batch to ensure an even distribution of each group/timepoint over the different batches. **(B)** Separation between cells positive and negative for each hashtag. **(C)** Number of hashtags identified in each cell. **(D)** Genotype consistency between samples of the same donor (TRUE) or different donor (FALSE). **(E)** Comparison of genotype profiles. **(F)** Resulting cell counts per donor using hashtags (y axis), SNPs (x axis), or both (diagonal). Numbers not in the diagonal or edges indicate the number of cells identified differently by both methods.

Aliquots of 100,000 cryopreserved total cells from digested nasal biopsies were thawed in prewarmed 10% FCS RPMI 1640 and spun down at 300 *g* for 10 min. Cells were resuspended in Cell Staining Buffer (BioLegend) containing TruStain FcX-receptor block (BioLegend) and incubated for 10 min on ice. TotalSeq cell hashing antibodies targeting CD293 and β2 microglobulin (B2M) were then added following the manufacturer's recommendations, along with anti-CD45-APC (HI30; BioLegend). After a 15-min incubation period on ice, the cells were washed with staining buffer and spun down at 300 × *g* for 10 min at 4°C. Supernatant was removed, cells were resuspended in staining buffer, and all samples were combined before adding 7AAD for exclusion of dead cells. Live, single CD45^+^ cells were sorted on a FACSAriaIII (BD Biosciences) into Eppendorfs containing 0.04% BSA PBS. As samples were pooled during the sort, we could not determine input for each individual sample. However, assuming an average of 16% live mononuclear cells on the day of collection (as determined from the CyTOF data), and an average of 85% live cells during sort, the average input per sample could be estimated as ∼13,500 cells per sample. Cells were delivered to the Leiden Genome Technology Centre for single-cell sequencing on the 10x Genomics Chromium system. A maximum of 30,000 cells, from 15 barcoded samples, were encapsulated in each individual run, for a total of five runs. Cells were assigned to a run with an equal distribution of AR patients/controls, before/after challenge samples and collection date (Fig. S5 A). Cells were loaded according to the standard protocol of the Chromium single cell 3′ kit at a maximum concentration of 1,000 cells/μl. Sequencing was performed on two lanes of an Illumina Hiseq 4,000 to obtain coverage of at least 30,000 reads/cell. Raw sequencing data is available through the National Institutes of Health Sequence Read Archive (accession number PRJNA982584).

### Transcriptomic data mapping cells to subjects

Each barcode log-counts distribution over all cells of a single run followed a bimodal shape with well separable peaks. The high-count peak was interpreted as the signal for a particular barcoded donor. For each sample a two-component Gaussian mixture model was fitted and a cell was assigned to a donor when it was at least three times more likely to be explained by the donor’s high-count peak than by the donor’s noise low-count peak (Fig. S5 B). The majority of the cells were assigned to a single donor only (Fig. S5 C). Cells that could not be assigned to a single donor were excluded from further analyses.

The barcode-based assignment was validated by investigating concordance between genotypes of samples assigned to the same donor but sequenced in different runs. In the pileup of pooled, aligned reads from all runs, genomic positions (single nucleotide polymorphisms) likely to differ between donors were identified. At each such position and separately in each cell, we generated the count of transcripts with the reference base and count of transcripts with the alternative base(s). Cumulated positional counts of cells were assigned to the same sample and sample genotypes were called. Concordances were calculated for all sample pairs and clear separation was observed for concordances expected to belong to the same donor vs. different donors (Fig. S5, E and F). Positional counts of cells assigned to the same donor across multiple runs were cumulated and genotypes of all donors were called. A genotype-based donor assignment was then performed by identifying the most likely genotype for each cell. Cells that were consistently assigned to the same donor by barcode-based and genotype-based methods were selected for further analysis (Fig. S5 F and Table S7).

### scRNA-Seq analysis

The five demultiplexed gene expression matrices were imported in R package Seurat v3, retaining only genes expressed in at least three cells, and merged into a combined Seurat object. Cells with <200 or >4,000 unique molecular identifiers or more than 25% of mitochondrial RNAs were filtered out to exclude dead cells, doublets not excluded by the debarcoding, or any remaining cell clumps. After filtering, a total of 46,238 cells were left for subsequent analysis. To identify shared clusters of cells collected from different treatments (before and after challenge) and groups (patient and control), the gene-barcode matrix was integrated according to the integration workflow (Stuart et al., 2019) using SCT-transform to normalize and scale the gene-expression and reciprocal principal component analysis instead of canonical correlation analysis for dimensionality reduction (with 30 principal components). SCT normalized data were then used for further analysis of generated clusters.

Next, principal component analysis and uniform manifold approximation and projection (UMAP) were performed and 30 principal components were used as input for graph-based clustering (resolution = 0.5). To assign the resulting 26 clusters to a cell type, differential gene expression was performed using the Wilcox test (FindAllMarkers function, logfc.threshold = 0.25, only.pos = TRUE, min.diff.pct = 0.2, assay = “SCT”). The top 10 gene markers of each cluster were displayed with the DotPlot function. The number of cells per cluster in each condition was also calculated (see Tables S8 and S9) and plotted using UMAP projection and “split.by” options in Seurat.

Clusters 4, 5,13,15,18, 22, and 23 showed a typical MPS-like signature and were subset from the rest. These clusters were reclustered taking only these clusters into account, but this had little effect on clusters 4 and 5, which we have subclustered and described further down below (Figs. 3 and 4). To assign them more precisely to canonical MPS subgroups, the average gene expression of each cluster was calculated and plotted with the pheatmap package, along with a collection of canonical markers from the literature. To have an unbiased approach, we also iterated the FindAllMarkers function on this specific subgroup (FindAllMarkers function, logfc.threshold = 0.25, min.diff.pct = 0.2, assay = “SCT”). FindAllMarkers compares each cluster to all the others, so in this case, a more specific comparison to MPS-only clusters will be performed. Top gene markers per cluster were generated and sorted by fold change values in descending order. Dot plots and heat maps (DoMultiBarHeatmap function, https://gitcrcm.marseille.inserm.fr/herault/scHSC_herault/blob/ecf93aa2d914d0b3bd508d066∼887717d78b771/R_src/DoMultipleBarHeatmap.R) were then generated from these lists with the Seurat R package.

Clusters 4 and 5 were shown to contain multiple subclusters and were therefore individually subset and reclustered: data were first rescaled using the SCTtransform function, and dimensionality reduction and clustering (res = 0.5) were performed as in the general workflow. DEGs between subclusters were then identified with the FindAllMarkers function without a min.diff.pct threshold to include genes that are expressed in multiple subclusters, but at different levels. ALOX15 positive cells (ALOX15_POS) within the cDC2 cell cluster (T5) were subset from ALOX15 negative cells (ALOX15_NEG) with the WhichCells function and a metadata field based on ALOX15 expression was added. DEGs between the two subsets were determined with the FindAllMarkers function.

Each MPS cluster was analyzed to identify DEGs between conditions in each cell type (cluster). A combined metadata field was added, containing the combination of the cluster, the group (patient/control), and the treatment (before/after challenge) information. In this way, the FindMarkers function in Seurat allowed the identification of DEGs genes between two different subgroups of the same cell type (e.g., “control_Before_4” and “control_After_4” indicate a comparison between the control group before and after challenge for cluster 4). The test used for analysis between before and after challenge was the linear regression method that allowed the inclusion of donor information as a latent variable, to correct for an uneven donor distribution in some of the clusters between time points. For this analysis, we used the RNA assay in the FindMarkers function.

### In vitro stimulation of nasal biopsies

Cryopreserved cells from digested nasal biopsies were thawed as stated before and allowed to rest for 4 h in RPMI1640 supplemented with 10% FBS. To determine cytokine secretion from MPS cells, we utilized IL-10 and TNFα cytokine secretion assays (Miltenyi Biotec) according to the manufacturer’s protocol. Subsequently, samples were split and stimulated with 0.1 µg/ml ultrapure LPS from *Escherichia coli* 0111:B4 (Invivogen) or not stimulated. We have chosen LPS as a stimulus, as previous publications (Alexander et al., 2021; Byrne and Reen, 2002) and experiments from our group have shown this to be a good inducer of IL-10 in blood monocytes. After culture, cells were harvested, stained with a panel of cell surface markers for MPS lineage, activation markers, tolerance markers, and secreted cytokines (Table S6), and analyzed using a five-laser Aurora (Cytek Biosciences). Results from three individual experiments were normalized by CytoNorm using OMIQ. UMAP dimensionality reduction and phenograph clustering on monocyte/DC lineage markers were performed on live CD3^−^/CD19^−^/CD56^−^/CD11c^+^ single cells using OMIQ.

### Gene Ontology (GO) enrichment analysis

Enrichr (Kuleshov et al., 2016) GO enrichment analysis was performed on the top 15 (as sorted by absolute log_2_ fold change) significantly DEGs (adjusted P value <0.05) of each major cluster within the MPS compartment (4, 5, 13, 15, 18, 22, 23) to determine the association with specific cell types based on annotations in the Human Cell Atlas and ARCHS4 tissue databases. The terms with the highest combined score (calculated by multiplying the log of the P value of the Fishers exact test by the *z* score of the deviation from the expected rank) were selected for visualization.

To phenotype/identify the individual cell clusters generated by subclustering the cDC2 (cluster 5) and CD14^+^ monocyte (cluster 4) populations, GO enrichment analysis of biological processes (BP), protein complex database (CORUM), and Reactome pathways was performed with g:profiler (Raudvere et al., 2019) on the top 40 (as sorted by absolute log_2_ fold change) significantly DEGs (adjusted P value <0.05) between subclusters. Genes were analyzed as an unordered query using g:profiler’s default g:SCS threshold-based methods to correct for multiple testing, and all annotated genes detected in the transcriptomic data as a background set. Lists of enriched GO categories were collapsed and simplified with Revigo (Supek et al., 2011) using the *Homo sapiens* set of GO terms. GO terms with dispensability values ≤0.5 were then visualized in a dot plot (ggplot function, RStudio version 4.0), depicting the negative-adjusted log_10_ P value and the gene ratio, which equals the number of DEGs against the number of genes associated with the GO term.

### Statistical analysis

Analysis of proteomic lymphocyte and granulocyte cell frequency data was performed with two-tailed, Wilcoxon matched-pairs signed rank tests, or Mann–Whitney test with a 95% confidence interval (Prism, GraphPad Software).

The normalized frequencies of the MPS cell clusters in both the transcriptomics and proteomic data were compared between non-allergic and allergic groups and between before and after allergen challenge. For this analysis, a generalized linear mixed model was fitted with an underlying binomial distribution. For each cluster, a separate model was fitted for the control group and the allergic group. The challenge was modeled as fixed effect. Lastly, volunteer ID was included as a random effect and a sample-specific random effect was included to deal with the overdispersion. Median fluorescence intensities were compared using a similar linear mixed model. Here a Gaussian distribution was assumed and as random effect only volunteer ID was included. Estimated marginal means were used for post-hoc analysis using the Emmeans package. To control the FDR, P values were corrected based on the Benjamini-Hochberg method, with P < 0.1 considered significant. *P < 0.05; **P < 0.01; ***P < 0.001. Median and interquartile ranges are shown in the bar graphs.

### Integration of datasets

To find features that are correlated between the CyTOF and scRNA-Seq datasets and predictive of outcome, we used the “Diablo” algorithm (Singh et al., 2019; mixOmics_v6.3.2, run in R v4.2.1) with a correlation strength between datasets of 1. This algorithm combines canonical correlation analysis and partial least squares discriminant analysis, with a Lass-like regularization to classify groups and identify the most important features. Of note, as granulocytes could not be analyzed by scRNA-Seq, we used CyTOF cluster frequencies from Live/CD45^+^/CD66b^−^ cells, except for the percentages of neutrophils, eosinophils, and basophils. Datasets were normalized per individual using the “withinVariation” function prior to use in Diablo to compare pre- and post-responses, and models were fitted for non-allergic controls and AR patients separately. The model performance was assessed with two components, and to select the number of components and features to retain, a leave-person-out cross-validation was performed with a range of feature numbers from two to six per dataset and component. Accuracy, as a percentage of correct predictions, was assessed using the “predict” function based on the “WeightedVote” output and centroids distance. The accuracy was assessed for both the first and second components. The number of features selected for the final model was based on the maximum accuracy per group, with the lowest number of features selected in case of models with similar accuracy.

### Receptor–ligand interactions

DEGs within clusters between non-allergic and AR groups were identified using the FindMarkers function of Seurat with the cut-off threshold of an adjusted P value smaller or equal to 0.05. DEGs were then used by the CellPhoneDB package v4 to determine relevant ligand–receptor interactions between clusters (Efremova et al., 2020). This analysis method only selects interactions where all the genes are expressed by at least 10% of the cells within a cluster and at least one gene is a DEG. CellPhoneDB calculates mean values based on the total mean of the individual partner average expression values in the corresponding interacting pairs of cell types. By running CellPhoneDB separately for the non-allergic and AR scRNA-Seq datasets, a log_2_ fold change between these interaction means per patient group could be calculated.

### Study approval

All subjects gave written informed consent and research was conducted in compliance with all relevant ethical regulations. Ethical approval was given by Erasmus Medical Centre Ethics Committee in Rotterdam (MEC2016-560).

### Online supplemental materials

Fig. S1 provides lineage marker expression on the UMAPs of CyTOF and scRNA-Seq data. Furthermore, it shows the UMAPs of the scRNA-Seq data for each group/timepoint. Fig. S2 provides the GO terms corresponding with CD14^+^ monocyte and cDC2 subcluster gene expression. Fig. S3 provides QC measures for scRNA-Seq data, cluster stability analysis, and contributions of each donor to each cluster. Fig. S4 provides expression of lineage markers and top differentially expressed markers on the cDC2 subclusters, the correlation between cluster frequencies as determined by scRNA-Seq and CyTOF as well as receptor–ligand interaction analysis. Fig. S5 provides details on the demultiplexing of scRNA-Seq data. Table S1 provides the CyTOF antibody panel. Tables S2, S3, S4, and S5 provide the top DEGs in the MPS cluster, in the CD14^+^ monocyte subclusters, in the MPS cluster in response to allergen challenge, and in the cDC2 subclusters, respectively. Table S6 provides the antibody panel used for the spectral cytometry analysis in Fig. 6. Tables S7, S8, and S9 provide for the scRNA-Seq data the number of cells identified in each cluster per donor, per group/timepoint per cluster, and for each donor/group/timepoint, respectively. Table S10 provides for the CyTOF data the number of cells identified in each cluster per donor/group/timepoint. Table S11 provides characteristics for each donor, including co-sensibilization.

## Supplementary Material

Table S1 provides mass cytometry antibody panel.

Table S2 provides top DEGs in MPS clusters.

Table S3 provides top DEGs in CD14^+^ subclusters.

Table S4 provides top DEGs in MPS in response to allergen challenge.

Table S5 provides top DEGs in cDC2 subclusters.

Table S6 provides spectral cytometry antibody panel.

Table S7 provides the number of cells per donor per cluster in transcriptomic data.

Table S8 provides the number of cells per group/timepoint per (sub)cluster in transcriptomic data.

Table S9 provides the number of cells per group/timepoint per (sub)cluster and donor in transcriptomic data.

Table S10 provides the number of cells in each (sub)cluster and donor for proteomic data.

Table S11 provides patient characteristics and co-sensitization.

## Data Availability

Patient characteristics, cells per cluster, and DEGs are available in the online supplemental material. The data underlying Fig. 1, Fig. 5, A–C, and Fig. S1 are openly available in the Flow Repository with Repository ID FR-FCM-Z69M. The data underlying Figs. 2, 3, and 4; Fig. 5, F and G; and Figs. S2, S3, S4, and S5 are deposited in the National Institutes of Health Sequence Read Archive (accession number PRJNA982584), where they are available upon request.
