# Correction: Inflammatory and tolerogenic myeloid cells determine outcome following human allergen challenge

**DOI:** 10.1084/jem.2022111108162023c

**Published:** 2023-08-18

**Authors:** Astrid L. Voskamp, Tamar Tak, Maarten L. Gerdes, Roberta Menafra, Ellen Duijster, Simon P. Jochems, Szymon M. Kielbasa, Tom Groot Kormelink, Koen A. Stam, Oscar R.J. van Hengel, Nicolette W. de Jong, Rudi W. Hendriks, Susan L. Kloet, Maria Yazdanbakhsh, Esther C. de Jong, Roy Gerth van Wijk, Hermelijn H. Smits

Vol. 220, No. 9 | https://doi.org/10.1084/jem.20221111 | July 10, 2023

The authors regret that Simon P. Jochems was inadvertently omitted from the author list in the original version of their article. The corrected author list appears above. The error appears in PDFs downloaded before August 16, 2023.

